# Analysis of the burden of colorectal cancer attributable to high body mass index in 204 countries and regions worldwide from 1990 to 2021

**DOI:** 10.3389/fnut.2025.1589250

**Published:** 2025-06-09

**Authors:** Mi Zhao, Ya Zheng, Zhaofeng Chen

**Affiliations:** 1 The First Clinical Medical College, Lanzhou University, Lanzhou, China; 2 Department of Gastroenterology, The First Hospital of Lanzhou University, Lanzhou, China; 3 Gansu Province Clinical Research Center for Digestive Diseases, The First Hospital of Lanzhou University, Lanzhou, China

**Keywords:** colorectal cancer, Global Burden of Disease, high body mass index, health inequality, socio-demographic Index

## Abstract

**Background:**

Colorectal cancer (CRC) is the second most common malignancy and the third leading cause of cancer-related deaths globally. Numerous studies have established a link between high body mass index (BMI) and CRC. However, a detailed analysis of the global disease burden of CRC attributable to high BMI has been lacking.

**Objective:**

This study aimed to evaluate the spatiotemporal trends in mortality and disability-adjusted life years (DALYs) attributable to high BMI-related CRC at global, regional, and national levels from 1990 to 2021.

**Methods:**

Epidemiological data on the association between high BMI and CRC were extracted from the 2021 Global Burden of Disease (GBD) study. Data on mortality, DALYs, age-standardized mortality rate (ASMR), and age-standardized DALY rate (ASDR) were stratified by sex, year, age, country, and Socio-demographic Index (SDI). Estimated annual percentage changes (EAPC) were calculated to assess temporal trends in ASMR and ASDR attributable to high BMI from 1990 to 2021. Decomposition and frontier analyses were conducted to identify drivers of burden changes and top-performing countries. Inequality analysis was performed to assess burden disparities across different SDI levels. The Bayesian age-period-cohort (BAPC) model was used to predict disease burden up to 2050.

**Results:**

Deaths and DALYs related to high BMI-associated CRC showed a robust upward trend, more than doubling in absolute numbers since 1990. Without intervention, similar patterns are projected to continue over the next 29 years. East Asia exhibited the highest risk of CRC deaths and DALYs attributable to high BMI, with the heaviest burden observed in China and the United States. High SDI regions demonstrated a higher burden, while low SDI regions faced higher EAPC.

**Conclusion:**

This study highlights high BMI as a significant risk factor for CRC, with notable regional heterogeneity in disease burden. Stratification by SDI and health inequality analysis underscore the need for tailored preventive strategies and health interventions targeting high BMI, particularly in different SDI regions.

## 1 Introduction

Colorectal cancer (CRC) represents a prevalent and clinically significant gastrointestinal malignancies, characterized by both high incidence rates and substantial mortality burden (1). In 2022, CRC ranked as the third most prevalent gastrointestinal malignancy worldwide, with approximately 1.9 million new cases (9.6% of total cancer cases), and the second leading cause of cancer-related mortality, accounting for nearly 903,859 deaths (9.3% of total cancer deaths) (2). Forecasts suggest the global incidence of colorectal cancer (CRC) is anticipated to surge to approximately 2.5 million new cases annually by 2035 (3). The development and progression of CRC are influenced by multiple risk factors, including population aging and dietary patterns in high-income countries, as well as modifiable factors such as obesity (elevated body mass index) and lack of physical exercise, which have been consistently associated with increased CRC risk (4, 5). Additionally, non-modifiable factors, including family history and specific genetic syndromes such as familial adenomatous polyposis, significantly contribute to elevated CRC susceptibility (6, 7).

High BMI, defined as a BMI of 25 kg/m^2^ or higher in individuals aged ≥20 years, is a well-established health risk associated with premature mortality. In recent years, the disease burden and prevalence attributable to elevated BMI have increased rapidly. High BMI contributes to a substantial burden of numerous chronic conditions, including cardiovascular diseases, type 2 diabetes, chronic kidney disease, and various types of cancer (8–10). Cancer, a group of diseases resulting from the complex genic interaction, environmental, and behavioral factors, is significantly influenced by high BMI, which ranks as the third leading risk factor for cancer development, following smoking and infections (11). Globally, over 400,000 cancer-related deaths in 2019 were attributable to high BMI (12). In Europe, approximately 11% of CRC cases are linked to overweight and obesity. Epidemiological evidence indicates that obesity increases the risk of colon cancer by 30–70% in men (13). The link between high BMI and colorectal cancer (CRC) has been extensively acknowledged, with a substantial number of CRC cases attributed to modifiable risk factors, notably high BMI (14, 15). Notably, the proportion of CRC cases attributable to high BMI rose to 7.8% in 2019 (16).

Although high BMI represents a modifiable risk factor, its impact on digestive system cancers, particularly in terms of CRC burden, remains underexplored. Previous GBD studies have provided an overview of CRC burden, analyzing disparities in incidence and mortality rates across regions and age groups. From 1990 to 2019, these studies also briefly examined risk factors associated with CRC (17). However, these analyses did not specifically investigate the burden of CRC attributable to high BMI.

The Global Burden of Diseases, Injuries, and Risk Factors (GBD) study serves as a comprehensive health data platform (18). Leveraging the latest data from GBD 2021, this study is designed to quantify the global, regional, and national burden of CRC attributable to high BMI. Additionally, we assess the potential correlation between the SDI and CRC burden, as well as project disease burden trends through 2050. By providing a deeper understanding of the impact of high BMI, this study seeks to advance epidemiological insights into high BMI-related CRC and inform targeted prevention strategies to mitigate the escalating burden of CRC.

## 2 Materials and methods

### 2.1 Data sources

All data utilized in this study were sourced from the GBD 2021 study (19, 20), which provides a comprehensive and systematic assessment of the global, regional, and national disease burden attributable to various risk factors, including high BMI, with specific attribution to conditions such as CRC. We extracted data on CRC-related deaths, ASMR, DALYs, and ASDR attributable to High BMI in 204 Countries and Regions Worldwide from 1990 to 2021. ASMR is a statistical measure that enables the comparison of mortality rates between populations with differing age structures by adjusting for age-related differences. Similarly, ASDR accounts for both mortality and disability while adjusting for age structure. Data retrieval was conducted through the online Global Health Data Exchange platform^1^.

The search criteria included “colorectal cancer” as the primary keyword, with “high BMI” selected from the risk factor list. We collected data on the absolute numbers and age-standardized rates (ASRs) of CRC attributable to high BMI across 21 regions and 204 countries.

### 2.2 Definitions

High BMI is classified for individuals aged 20 years or older with a BMI exceeding 25 kg/m^2^ (19, 21). The SDI is a composite metric that evaluates the average per capita income, fertility rate, and educational attainment across regions and countries. The SDI scale ranges from 0 to 1, with 0 representing the lowest and 1 representing the highest level of development. Based on SDI values, regions and countries are categorized into five distinct groups: high SDI (>0.81), high-middle SDI (0.70–0.81), middle SDI (0.61–0.69), low-middle SDI (0.46–0.60), and low SDI (<0.46) (21).

### 2.3 Statistical analysis

#### 2.3.1 Burden description

This study compiled data from 21 geographical regions and 204 countries/territories worldwide, encompassing absolute case numbers and age-standardized incidence rates (ASRs) of colorectal cancer (CRC) attributable to high BMI. Additionally, decomposition, frontier, and health inequality analyses were conducted. Finally, a BAPC model was employed to project changes in disease burden through 2050.

#### 2.3.2 Decomposition analysis

We applied Das Gupta’s decomposition method to quantify the contributions of age structure, epidemiological changes, and population growth to the burden of CRC attributable to high BMI (22–24). Epidemiological dynamics, reflected in changes in disease incidence and case-fatality rates, represent the outcomes of advancements in medical science and public health interventions. Population growth, a critical driver of disease burden, can exacerbate the burden even if incidence and case-fatality rates remain stable. Population aging, characterized by an increasing proportion of elderly individuals, is likely to amplify the burden of chronic and non-communicable diseases.

#### 2.3.3 Age-period-cohort analysis

The age-period-cohort (APC) model was used to evaluate the dynamic effects of age, period, and cohort factors on disease outcomes. Based on a Poisson distribution, the APC model decomposes variables into age, period, and cohort dimensions to analyze their influence on the risk of CRC incidence and mortality associated with high BMI (25).

#### 2.3.4 Frontier analysis

Frontier analysis, a key quantitative tool in GBD studies, employs data envelopment analysis (DEA) and locally estimated scatterplot smoothing (LOESS) to assess and optimize health system performance. This method is particularly valuable for examining the relationship between disease burden and socio-demographic development. By constructing a “frontier” or best-practice boundary, frontier analysis identifies the minimum achievable age-standardized disease burden at different SDI levels.

#### 2.3.5 Projection analysis

This study employed a BAPC model to project the colorectal cancer burden attributable to high BMI from 2022 to 2050. The model assumes a Poisson distribution for the data and incorporates three random effects: age, period, and cohort (modeled via a second-order random walk, rw2), along with an additional independent and identically distributed (iid) random effect to adjust for overdispersion. Prior to model implementation, performance was rigorously evaluated using three metrics: coverage (calculated as the proportion of projections within the 95% credibility interval), bias (assessed by comparing observed incidence rates to predicted values), and precision (quantified using posterior standard deviations). To comprehensively characterize uncertainty in projections, we further provide numerical estimates, standard deviations, and prediction intervals (50–95%) for the projection period spanning 2022–2050. These results enable robust quantification of uncertainty and enhance the interpretability of long-term burden forecasts (26, 27).

#### 2.3.6 Health inequality analysis

We employed the slope index of inequality (SII) and concentration index (CI) to quantify health inequalities in the burden of CRC attributable to high BMI across 204 countries and territories from 1990 to 2021 (28). The SII, a quantitative measure derived from regression analysis, assesses absolute differences in health outcomes across socioeconomic gradients (e.g., income, SDI). The CI evaluates the concentration of health outcomes across socioeconomic distributions. Both indices were used to analyze inequalities in disease burden (including DALY rates and DALYs) across regions, socioeconomic levels, age groups, and genders, with higher values indicating greater inequality.

#### 2.3.7 Statistical analysis

This study focused on estimating and presenting the ASMR and ASDR per 100,000 population for CRC attributable to high BMI, along with their corresponding 95% uncertainty intervals (UIs). As standardized measures of disease burden, ASMR and ASDR enable robust comparisons of disease burden across countries and over time. All statistical analyses and data visualizations were performed using the Health Equity Assessment Toolkit (HEAT), developed by the World Health Organization (WHO) and implemented in R software (version 4.4.1).

## 3 Results

### 3.1 Global and regional burden of CRC attributable to high BMI

At the global level, the number of deaths attributable to high BMI-related CRC increased from 41,536 cases (95% UI: 17,666–67,379) in 1990 to 99,268 cases (95% UI: 42,956–157,949) in 2021 (Table 1). Similarly, the number of DALYs rose from 1,015,042 (95% UI: 429,787–1,631,974) in 1990 to 2,364,664 (95% UI: 1,021,594–3,752,340) in 2021 (Table 2). In 2021, the ASMR and ASDR were 1.17 (95% UI: 0.51–1.87) and 27.33 (95% UI: 11.80–43.37) per 100,000 population, respectively. Temporal trend analysis revealed an increase in ASMR but a slight decline in ASDR for high BMI-related CRC, with EAPCs of 0.12 (95% CI: 0.08–0.16) and −0.0014 (95% CI: −0.0418–0.0390), respectively (Tables 1, 2).

**Table 1:** Deaths and ASMR of CRC attributable to the high BMI in 1990 and 2021 and the EAPC from 1990 to 2021.

Deaths	1990		2021		EAPC (1990–2021)
Location	Death cases (95%UI)	ASMR (95%UI)	Death cases (95%UI)	ASMR (95%UI)	ASMR (95%CI)
Global	41535.76 (17665.60,67379.01)	1.14 (0.48,1.86)	99267.99 (42956.34,157948.81)	1.17 (0.51,1.87)	−0.00 (−0.04,0.04)
High SDI	21852.17 (9256.42,35533.19)	1.96 (0.83,3.19)	36529.90 (15670.33,58138.72)	1.68 (0.73,2.66)	−0.64 (−0.69,−0.59)
High-middle SDI	13679.92 (5864.87,22167.68)	1.44 (0.61,2.33)	32966.43 (14288.45,52368.09)	1.67 (0.72,2.66)	0.40 (0.32,0.48)
Middle SDI	4237.34 (1566.48,6974.55)	0.43 (0.16,0.70)	21653.80 (9249.92,34503.05)	0.82 (0.35,1.32)	2.13 (2.11,2.16)
Low-middle SDI	1244.46 (472.06,1981.84)	0.21 (0.08,0.33)	6391.15 (2689.53,10103.36)	0.45 (0.19,0.71)	2.72 (2.65,2.80)
Low SDI	442.33 (161.41,737.04)	0.20 (0.07,0.33)	1567.46 (614.91,2540.44)	0.32 (0.12,0.52)	1.50 (1.38,1.63)
Andean Latin America	134.42 (54.90,221.17)	0.67 (0.28,1.12)	672.35 (287.38,1130.55)	1.15 (0.49,1.93)	1.81 (1.68,1.94)
Australasia	560.56 (233.15,893.87)	2.41 (1.00,3.83)	1123.44 (476.29,1796.53)	2.01 (0.85,3.20)	−0.78 (−0.87,−0.70)
Caribbean	243.85 (101.78,387.50)	0.97 (0.40,1.54)	841.41 (355.22,1385.03)	1.55 (0.66,2.56)	1.68 (1.62,1.73)
Central Asia	488.23 (201.65,783.84)	1.04 (0.43,1.68)	768.89 (328.84,1215.08)	0.96 (0.41,1.53)	0.10 (−0.03,0.22)
Central Europe	3627.88 (1590.10,5836.46)	2.46 (1.08,3.96)	6940.62 (3093.02,11133.30)	3.03 (1.35,4.85)	0.56 (0.42,0.70)
Central Latin America	529.59 (223.35,852.82)	0.67 (0.28,1.07)	3148.92 (1402.87,5105.41)	1.26 (0.56,2.06)	2.11 (2.03,2.19)
Central Sub-Saharan Africa	50.43 (18.92,86.22)	0.23 (0.09,0.39)	254.12 (96.36,437.84)	0.49 (0.18,0.83)	2.41 (2.24,2.58)
East Asia	3773.04 (1309.29,6405.90)	0.45 (0.16,0.77)	20370.79 (8474.68,33798.87)	0.96 (0.40,1.59)	2.41 (2.34,2.48)
Eastern Europe	5380.98 (2331.99,8579.24)	1.92 (0.83,3.06)	9051.62 (3878.38,14440.41)	2.53 (1.09,4.04)	0.72 (0.60,0.85)
Eastern Sub-Saharan Africa	208.54 (73.42,349.52)	0.28 (0.10,0.46)	773.78 (295.18,1284.42)	0.48 (0.18,0.80)	1.62 (1.52,1.72)
High-income Asia Pacific	1424.94 (521.28,2304.16)	0.73 (0.27,1.17)	4240.01 (1633.47,6743.66)	0.83 (0.32,1.31)	0.32 (0.27,0.36)
High-income North America	8084.09 (3450.06,13011.39)	2.27 (0.97,3.65)	12769.32 (5673.42,19869.58)	1.95 (0.87,3.03)	−0.66 (−0.77,−0.55)
North Africa and Middle East	1341.11 (566.00,2184.21)	0.83 (0.35,1.35)	5636.83 (2450.65,8982.28)	1.31 (0.58,2.10)	1.70 (1.53,1.86)
Oceania	14.67 (5.94,24.54)	0.51 (0.21,0.85)	43.47 (18.02,70.34)	0.59 (0.25,0.96)	0.51 (0.41,0.61)
South Asia	516.89 (173.35,827.31)	0.09 (0.03,0.14)	3042.70 (1182.31,4777.43)	0.20 (0.08,0.32)	2.81 (2.76,2.86)
Southeast Asia	652.61 (220.65,1050.34)	0.25 (0.08,0.40)	4057.28 (1622.97,6569.23)	0.62 (0.25,1.00)	3.04 (2.93,3.15)
Southern Latin America	932.69 (403.02,1520.71)	2.07 (0.89,3.36)	2235.24 (992.44,3641.51)	2.52 (1.12,4.11)	0.90 (0.72,1.09)
Southern Sub-Saharan Africa	197.15 (83.48,309.96)	0.77 (0.33,1.22)	807.85 (341.44,1269.91)	1.49 (0.63,2.34)	2.32 (2.05,2.60)
Tropical Latin America	699.20 (289.79,1135.93)	0.80 (0.33,1.29)	3629.77 (1533.54,5850.43)	1.42 (0.60,2.29)	1.87 (1.75,1.98)
Western Europe	12481.11 (5253.07,20401.79)	2.11 (0.89,3.45)	18025.55 (7622.60,29686.28)	1.77 (0.75,2.90)	−0.65 (−0.72,−0.59)
Western Sub-Saharan Africa	193.79 (76.49,312.94)	0.24 (0.09,0.38)	834.03 (336.27,1365.09)	0.47 (0.19,0.75)	2.39 (2.33,2.45)

**Table 2:** DALYs and ASDR of CRC attributable to the high BMI in 1990 and 2021 and the EAPC from 1990 to 2021.

DALYs	1990		2021		EAPC (1990–2021)
Location	DALYs cases (95%UI)	ASDR (95%UI)	DALYs cases (95%UI)	ASDR (95%UI)	ASDR (95%CI)
Global	1015042.12 (429787.23,1631973.77)	25.54 (10.83,41.20)	2364664.16 (1021593.57,3752340.44)	27.33 (11.80,43.37)	0.12 (0.08,0.16)
High SDI	490182.58 (210224.59,788176.56)	44.94 (19.27,72.19)	775808.59 (337834.35,1225933.91)	40.00 (17.48,62.93)	−0.48 (−0.52,−0.43)
High-middle SDI	346229.15 (147488.85,559452.79)	34.20 (14.56,55.28)	769289.88 (332394.97,1220597.72)	39.23 (16.94,62.34)	0.31 (0.24,0.38)
Middle SDI	125727.81 (46880.38,206617.42)	11.03 (4.10,18.13)	584511.44 (248469.11,929607.81)	21.01 (8.93,33.45)	2.10 (2.06,2.14)
Low-middle SDI	37581.20 (14268.97,60129.19)	5.52 (2.10,8.81)	184447.78 (77501.94,290094.43)	11.87 (5.00,18.73)	2.69 (2.62,2.76)
Low SDI	13400.07 (4984.01,22374.67)	5.28 (1.94,8.78)	47046.61 (18740.19,75740.41)	8.17 (3.23,13.20)	1.33 (1.21,1.46)
Andean Latin America	3674.46 (1506.46,5995.46)	16.83 (6.89,27.44)	16947.37 (7443.67,28548.21)	28.00 (12.28,47.21)	1.67 (1.54,1.80)
Australasia	13424.06 (5579.52,21235.98)	58.17 (24.18,91.85)	23565.43 (10170.01,37480.47)	46.65 (20.18,74.39)	−0.94 (−1.03,−0.85)
Caribbean	6308.39 (2656.46,10111.52)	23.81 (10.00,38.23)	20386.21 (8687.12,33782.84)	37.94 (16.17,62.94)	1.66 (1.60,1.71)
Central Asia	14011.57 (5760.36,22453.15)	28.36 (11.66,45.42)	21393.21 (9092.71,33879.79)	24.46 (10.40,38.70)	−0.22 (−0.32,−0.13)
Central Europe	87450.39 (38342.25,140810.88)	57.78 (25.32,93.00)	148738.23 (66397.22,238495.58)	68.94 (30.82,110.60)	0.48 (0.34,0.62)
Central Latin America	14473.39 (6123.86,23290.96)	16.13 (6.82,25.96)	84536.93 (37821.77,134896.19)	32.79 (14.66,52.33)	2.32 (2.25,2.40)
Central Sub-Saharan Africa	1494.11 (554.64,2559.87)	5.96 (2.23,10.14)	7638.09 (2874.63,13167.73)	12.19 (4.62,21.03)	2.34 (2.18,2.50)
East Asia	113878.45 (39419.44,193735.90)	11.93 (4.13,20.29)	529351.86 (219132.65,886681.30)	24.42 (10.09,40.83)	2.31 (2.21,2.41)
Eastern Europe	137909.20 (59624.98,219010.62)	48.61 (20.98,77.14)	208723.80 (90209.85,331586.18)	60.00 (25.94,95.33)	0.45 (0.31,0.59)
Eastern Sub-Saharan Africa	6358.76 (2271.42,10621.76)	7.52 (2.67,12.61)	23026.17 (8703.46,37648.98)	11.97 (4.57,19.80)	1.33 (1.23,1.44)
High-income Asia Pacific	36685.59 (13456.32,59450.74)	17.92 (6.55,28.98)	80601.36 (31642.07,127988.08)	19.32 (7.65,30.53)	0.13 (0.09,0.18)
High-income North America	185196.87 (80214.15,295439.61)	54.48 (23.66,86.76)	296819.56 (135095.20,460708.66)	49.52 (22.63,76.52)	−0.42 (−0.51,−0.33)
North Africa and Middle East	39567.66 (16500.49,65046.47)	21.25 (8.93,34.73)	156482.30 (66565.01,247801.81)	31.83 (13.68,50.63)	1.46 (1.31,1.60)
Oceania	470.59 (193.93,787.70)	13.60 (5.52,22.78)	1362.00 (562.24,2191.09)	15.48 (6.43,25.08)	0.44 (0.37,0.52)
South Asia	16693.79 (5702.77,26476.16)	2.47 (0.83,3.93)	91500.24 (35882.98,144388.46)	5.67 (2.22,8.92)	2.74 (2.69,2.79)
Southeast Asia	20952.73 (7310.93,33667.86)	7.05 (2.42,11.33)	119340.38 (48437.38,193338.90)	16.64 (6.73,26.92)	2.84 (2.71,2.97)
Southern Latin America	22071.59 (9492.58,36141.28)	47.48 (20.42,77.60)	50169.57 (22345.67,81467.57)	58.65 (26.14,95.15)	0.95 (0.79,1.12)
Southern Sub-Saharan Africa	5488.17 (2315.61,8602.25)	18.78 (7.96,29.48)	21915.39 (9192.30,34323.01)	35.73 (15.05,55.83)	2.36 (2.09,2.63)
Tropical Latin America	19341.38 (8091.73,31333.82)	19.74 (8.20,31.96)	94304.09 (39800.44,150657.52)	36.04 (15.20,57.63)	1.89 (1.78,2.00)
Western Europe	264280.07 (111568.97,431654.15)	46.80 (19.73,76.51)	344975.98 (147806.60,564627.85)	39.08 (16.77,63.51)	−0.67 (−0.74,−0.60)
Western Sub-Saharan Africa	5310.90 (2112.95,8558.91)	5.71 (2.26,9.23)	22886.00 (9127.63,37858.78)	10.79 (4.35,17.69)	2.20 (2.14,2.26)

In 2021, high-SDI regions exhibited the highest burden of CRC attributable to high BMI, with the largest number of deaths (36,530 cases; 95% UI: 15,670–58,139) and DALYs (775,809 cases; 95% UI: 337,834–1,225,934) among the five SDI regions. The ASMR and ASDR in high-SDI regions were also the highest, at 1.68 (95% UI: 0.73–2.66) and 40.00 (95% UI: 17.48–62.93) per 100,000 population, respectively. However, temporal trend analysis revealed a decline in ASMR and ASDR in high-SDI regions, whereas increases were observed in high-middle, middle, low-middle, and low-SDI regions.

At the regional level, East Asia had the highest number of deaths (20,371 cases; 95% UI: 8,475–33,799) and DALYs (529,352 cases; 95% UI: 219,133–886,681) attributable to high BMI-related CRC in 2021 among the 21 GBD regions. In contrast, Oceania recorded the lowest number of deaths (43 cases; 95% UI: 18–70) and DALYs (1,362 cases; 95% UI: 562–2,191). Central Europe exhibited the highest ASMR and ASDR at 3.03 (95% UI: 1.35–4.85) and 68.94 (95% UI: 30.82–110.60) per 100,000 population, respectively, while South Asia had the lowest ASMR and ASDR at 0.20 (95% UI: 0.08–0.32) and 5.67 (95% UI: 2.22–8.92) per 100,000 population, respectively.

From 1990 to 2021, Southeast Asia experienced the largest increases in ASMR and ASDR, with EAPCs of 3.04 (95% CI: 2.93–3.15) and 2.84 (95% CI: 2.71–2.97), respectively. Conversely, Australasia showed the most significant declines in ASMR and ASDR, with EAPCs of −0.78 (95% CI: −0.87 to −0.70) and −0.94 (95% CI: −1.03 to −0.85), respectively (Tables 1, 2 and Supplementary Figures S1, S2).

### 3.2 National burden of CRC attributable to high BMI

At the national level, China and the United States were the two countries with the highest number of deaths attributable to high BMI-related CRC in 2021, with 19,418 cases (95% UI: 8,053–32,452) and 11,402 cases (95% UI: 5,070–17,661), respectively. These were followed by the Russian Federation (6,486 cases; 95% UI: 2,777–10,321) and Germany (3,630 cases; 95% UI: 1,500–6,089). A similar pattern was observed for DALYs, with China, the United States, the Russian Federation, and Brazil ranking as the top four countries. The respective DALY counts were 507,316 (95% UI: 209,264–853,770) for China, 268,296 (95% UI: 122,073–412,891) for the United States, 147,446 (95% UI: 63,131–233,863) for the Russian Federation, and 92,420 (95% UI: 39,036–147,506) for Brazil.

**Figure 1:**
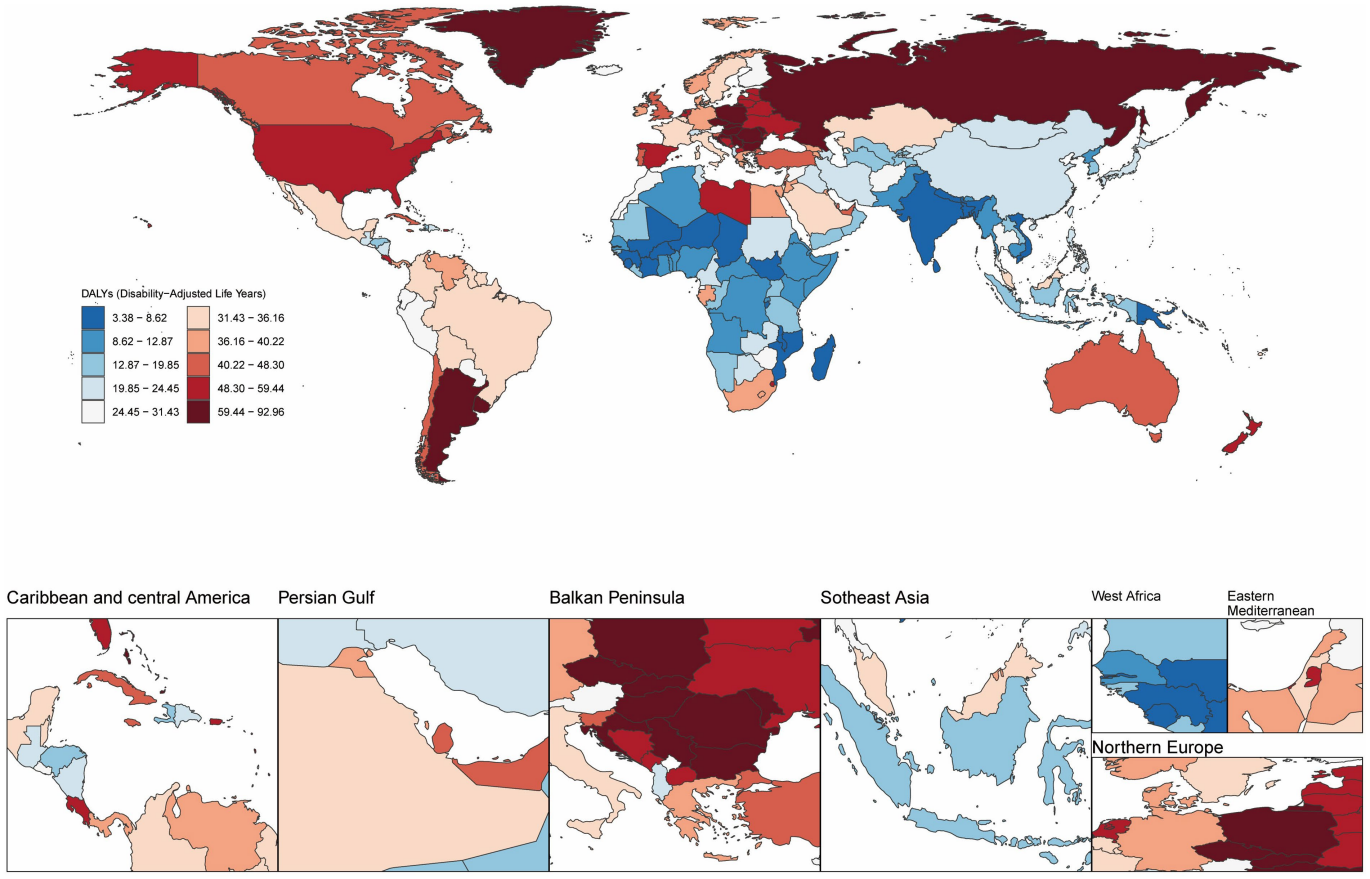
Age-standardized disability-adjusted life year rate (ASDR) per 100,000 population attributable to high BMI for CRC, by country/region, 2021.

Furthermore, in 2021, Hungary exhibited the highest ASMR and ASDR for high BMI-related CRC, at 3.79 (95% UI: 1.72–6.19) and 92.03 (95% UI: 41.88–147.88) per 100,000 population, respectively. Countries with the next highest ASMR were Slovakia, Uruguay, and Croatia, while Slovakia, Bulgaria, and Uruguay led in ASDR. From 1990 to 2021, Vietnam experienced the largest increases in ASMR and ASDR, with EAPCs of 4.65 (95% CI: 4.48–4.81) and 4.74 (95% CI: 4.53–4.95), respectively. In contrast, Austria showed the most significant declines in ASMR and ASDR, with EAPCs of −1.86 (95% CI: −1.92 to −1.80) and −1.88 (95% CI: −1.94 to −1.81), respectively (Figure 1; Supplementary Figure S3; Supplementary Tables S1, S2).

### 3.3 Age- and sex-specific burden of colorectal cancer attributable to high BMI

**Figure 2:**
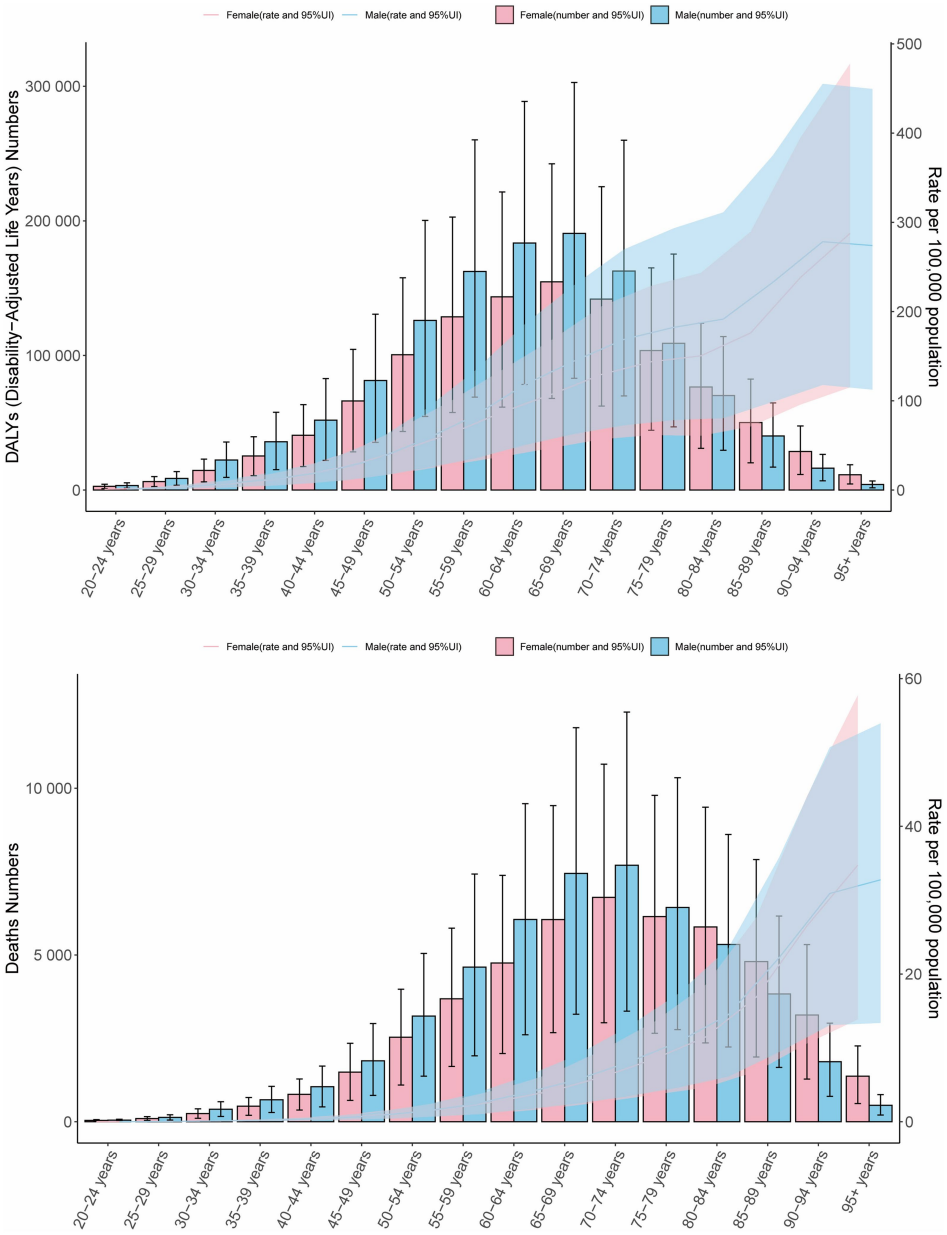
Age- and sex-specific DALY counts and rates, and mortality numbers and rates of CRC attributable to high BMI in 2021. Bars represent attributable burden, lines show age-specific rates by sex, and shaded areas denote 95% UIs.

In 2021, globally, age-specific mortality rates and DALY rates for CRC attributable to high BMI increased across all age groups in females. Males exhibited similar mortality and DALY patterns to females, except for individuals aged 90 years and older. Across all age groups, males demonstrated higher mortality and DALY rates than females. For both sexes, the number of deaths peaked in the 70–74 age group, while DALYs reached their highest level in the 65–69 age group. Except for the age group above 80 years, males consistently had higher numbers of deaths and DALYs than females in all other age groups (Figure 2).

Across SDI regions from 1990 to 2021, the trends in ASMR and ASDR for both sexes in high and high-middle SDI regions aligned with global patterns. In middle, low-middle, and low SDI regions, ASMR and ASDR for both sexes showed an increasing trend over time, whereas high SDI regions experienced a decline in ASMR and ASDR for both sexes. In high and high-middle SDI regions, males bore a higher burden of ASMR and ASDR for high BMI-related CRC compared to females, while the opposite was observed in low and low-middle SDI regions (Supplementary Figure S4).

### 3.4 Association between high BMI-related colorectal cancer and socio-demographic index (SDI)

**Figure 3:**
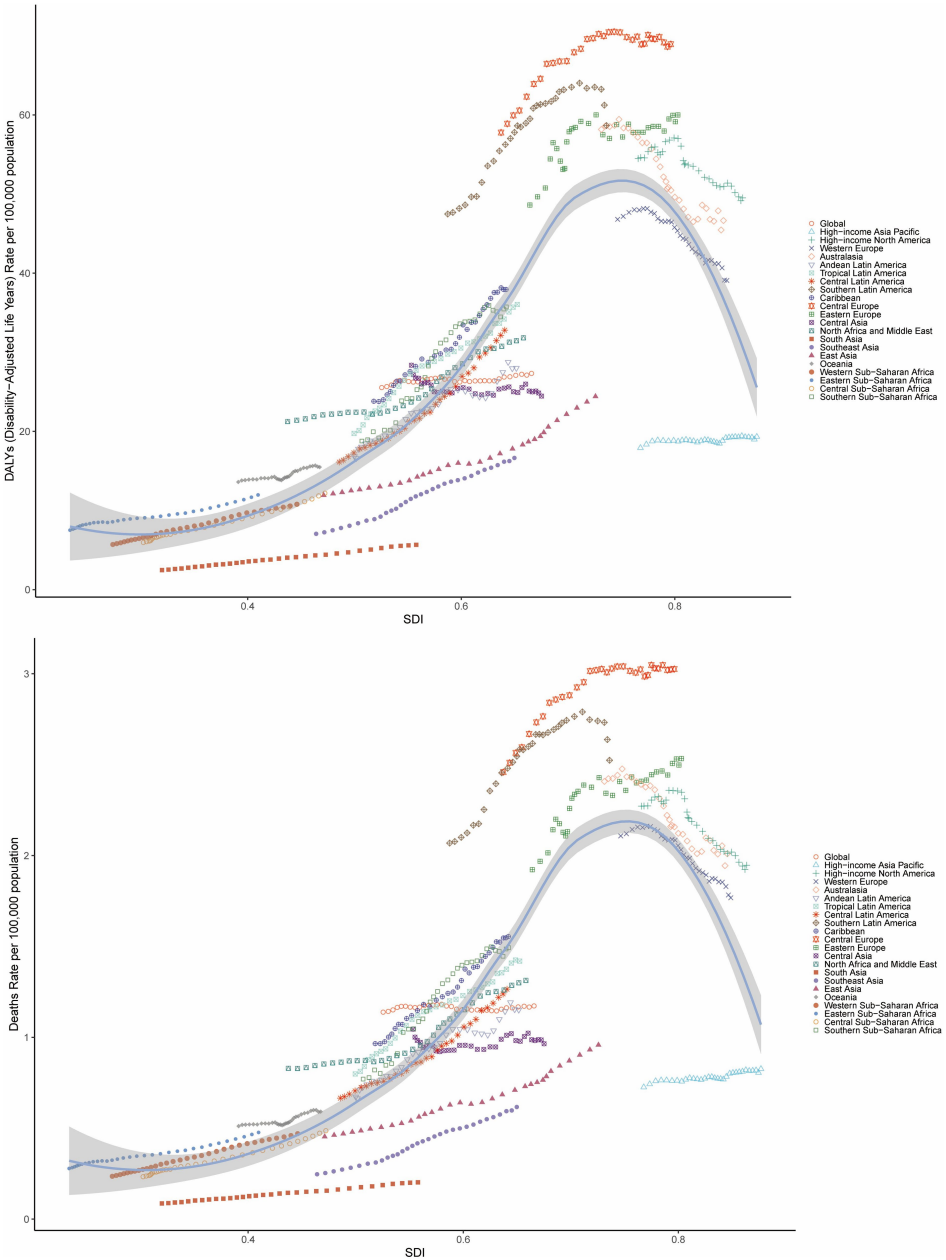
Age-standardized DALY rate and ASMR of CRC attributable to high BMI by SDI across 21 GBD regions, 1990–2021.

Analyses revealed a statistically significant non-linear association between the SDI and both ASMR and ASDR for BMI-attributable colorectal cancer across global and subregional GBD populations. Specifically, from 1990 to 2021, ASMR and ASDR for high BMI-related CRC in the 21 GBD regions initially increased with rising SDI, peaking at an SDI of approximately 0.75, after which the burden gradually declined with further increases in SDI. Notably, regions such as Central Europe, Southern Latin America, Eastern Europe, Australasia, and High-income North America exhibited higher ASMR and ASDR than expected for their SDI levels (Figure 3).

Globally, the correlation between age-standardized mortality rate (ASMR) and age-standardized disability rate (ASDR) for colorectal cancer attributable to high BMI and the Sociodemographic Index (SDI) exhibits distinct regional patterns. These metrics demonstrate a progressive increase until SDI reaches approximately 0.8, beyond which a downward trend in rates becomes evident (Supplementary Figure S5).

**Figure 4:**
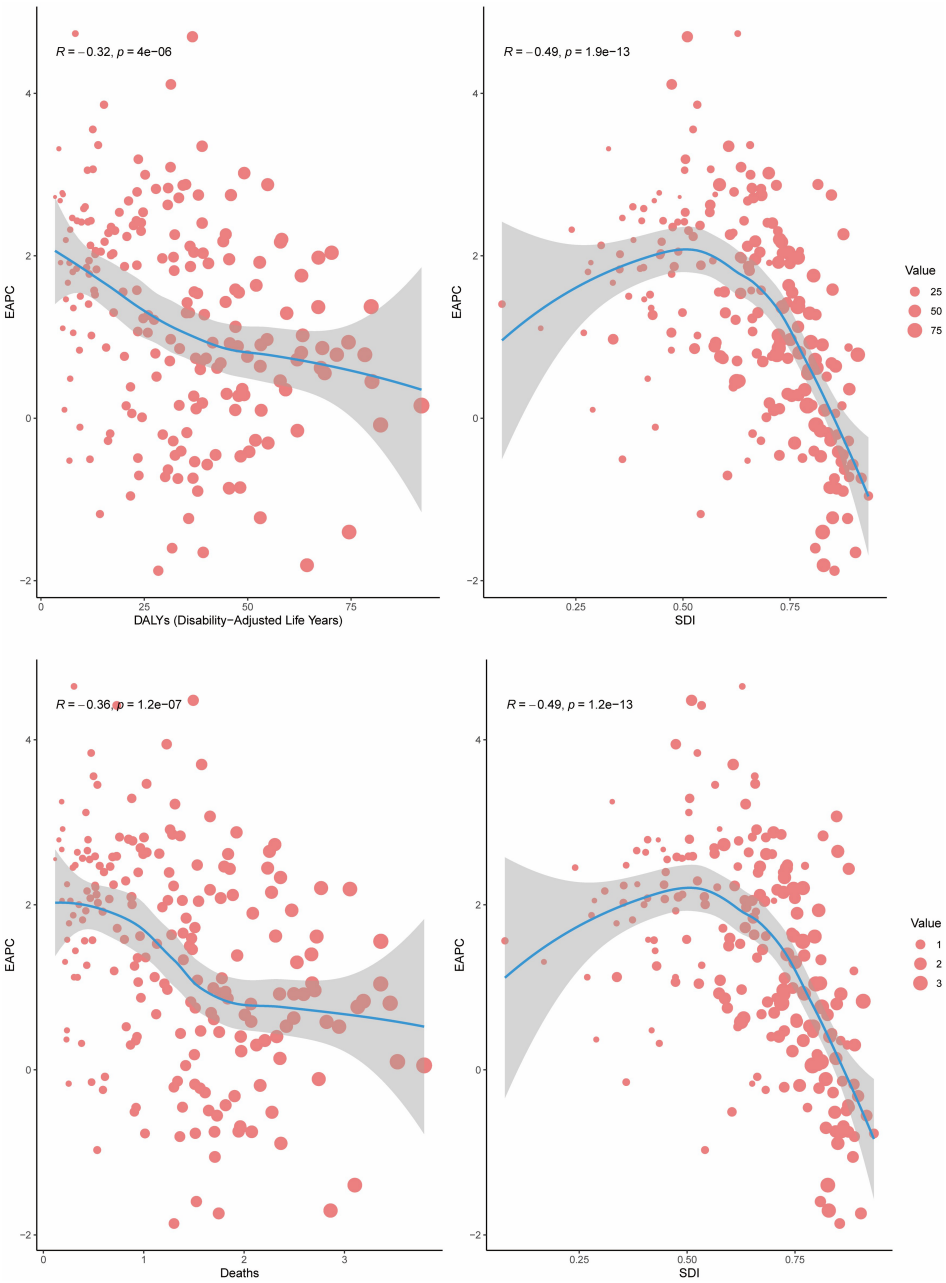
The correlation between the EAPC and ASDR/ASMR or SDI in 2021.

In 2021, a negative correlation was observed between the EAPC of mortality and DALYs and the ASMR or ASDR (*R* = −0.36 and −0.32, respectively; *p* < 0.001). Furthermore, for regions with SDI > 0.50, the EAPC of mortality and DALYs decreased with increasing SDI, demonstrating a significant negative correlation (*R* = −0.49 and −0.49, respectively; *p* < 0.001). This indicates that countries with higher SDI levels experienced a gradual decline in the burden of CRC attributable to high BMI (Figure 4).

### 3.5 Age-period-cohort analysis of the burden of colorectal cancer attributable to high BMI

**Figure 5:**
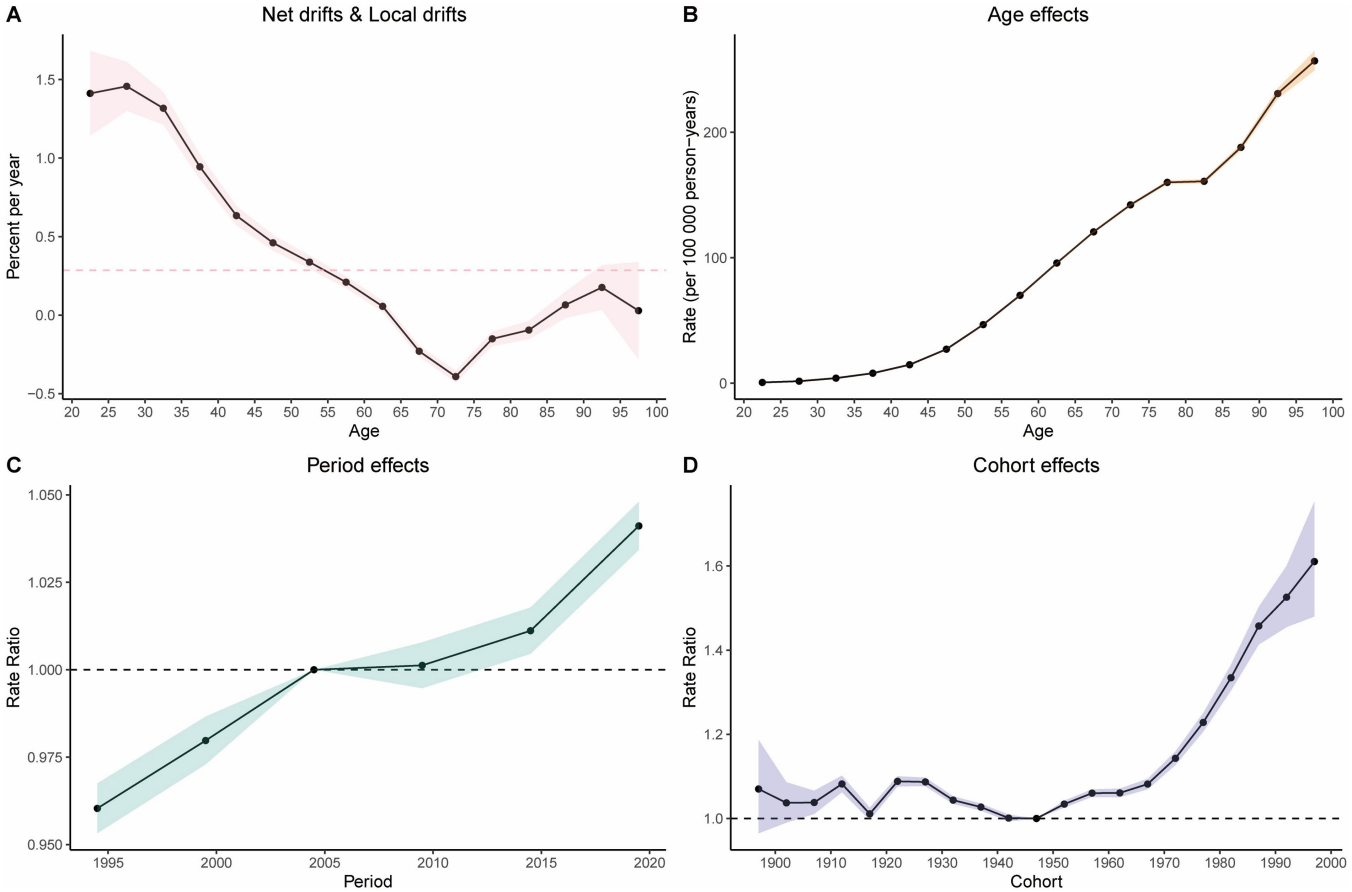
Results of the age-period-cohort analysis. **(A)** Net drift versus actual drift of disability-adjusted life years (DALYs). **(B)** Age effects on DALYs. **(C)** Period effects on DALYs. **(D)** Cohort effects on DALYs.

The age-period-cohort (APC) analysis of DALYs and mortality attributable to high BMI-related CRC revealed consistent trends (Figure 5 and Supplementary Figure S6). Age effect analysis: the age effect analysis demonstrated a significant upward trend in the burden of CRC attributable to high BMI with increasing age (Figure 5B). Period effect analysis: the period effect analysis indicated a gradual increase in the burden of high BMI-related CRC over time, except for a slight decline between 2005 and 2010 (Figure 5C). Cohort effect analysis: the cohort effect analysis showed that later-born cohorts experienced a significantly higher burden of CRC attributable to high BMI compared to earlier-born cohorts (Figure 5D). Net drift and local drift: net drift and local drift analyses further validated the dynamic changes in disease risk with age, revealing the annualized trends in DALY rates over time across different age groups. Specifically, the rate of change in DALYs decreased steadily until the 70–75 age group, reaching its minimum, after which it began to rise again (Figure 5A). The results of the APC analysis for mortality are presented in the supplementary materials (Supplementary Figure S6).

### 3.6 Decomposition analysis of the burden of CRC attributable to high BMI

We conducted a decomposition analysis to further explore the contributions of population aging, population growth, and epidemiological changes to the burden of CRC deaths and DALYs attributable to high BMI. The decomposition analysis revealed consistent patterns of contribution across global, five SDI regions, and 21 GBD regions.

**Figure 6:**
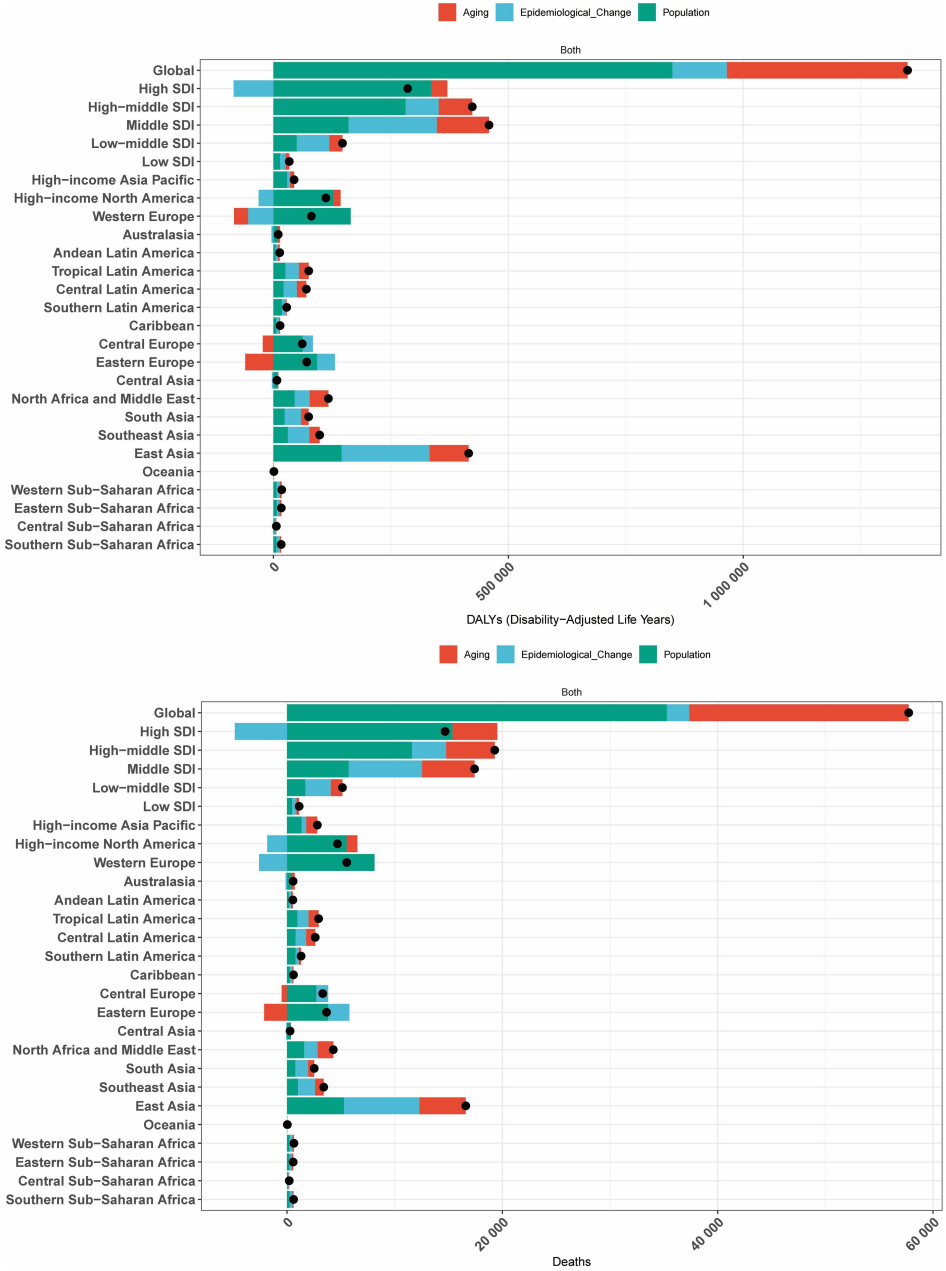
Decomposition analysis of changes in the DALYs and deaths of GBTCs attributable to high BMI across 21 GBD regions from 1990 to 2021.

Specifically, epidemiological changes in high SDI regions, Western Europe, and high-income North America reduced the burden of CRC attributable to high BMI. In Central Europe and Eastern Europe, population aging significantly decreased the burden of CRC attributable to high BMI, while in Western Europe, aging also contributed to a reduction in DALYs. In all other regions, population growth, aging, and epidemiological changes collectively increased the burden of CRC attributable to high BMI. Globally, population growth emerged as the primary driver of the increasing burden of CRC attributable to high BMI (Figure 6).

### 3.7 Health inequality analysis of the burden of CRC attributable to high BMI

**Figure 7:**
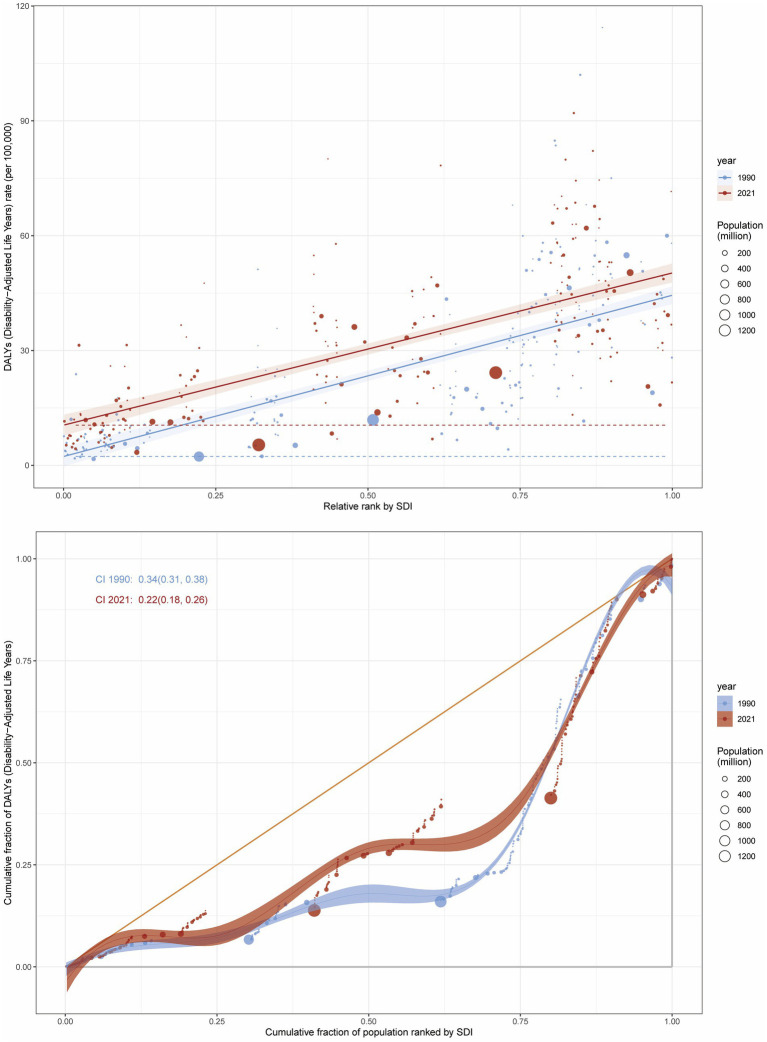
Inequality analysis of DALY attributable to high BMI in CRC at the global level in 1990 and 2021.

Pronounced socioeconomic disparities in both relative and absolute measures were evident within the high BMI-associated colorectal cancer burden, as quantified by the slope index of inequality (SII), which demonstrated a progressive rise between 1990 and 2021. Disproportionately higher burdens of CRC attributable to high BMI were observed in regions with higher SDI levels. The disparity in disease burden per 100,000 population between the highest and lowest SDI countries was 42.12 (95% CI: 37.25–47.00) in 1990 and 39.76 (95% CI: 34.36–45.15) in 2021 (Figure 7).

Additionally, the concentration index (CI) decreased from 0.34 (95% CI: 0.31–0.38) in 1990 to 0.22 (95% CI: 0.18–0.26) in 2021, indicating persistent inequalities between low and high SDI regions, although the relative concentration of the burden showed a decline (Figure 7). Similarly, for CRC mortality attributable to high BMI, the SII decreased from 1.80 (95% CI: 1.59–2.01) in 1990 to 1.77 (95% CI: 1.55–1.99) in 2021, while the CI declined from 0.37 (95% CI: 0.33–0.40) in 1990 to 0.24 (95% CI: 0.20–0.28) in 2021 (Supplementary Figure S7).

### 3.8 Frontier analysis of the burden of colorectal cancer attributable to high BMI

Frontier analysis demonstrates significant advantages and utility in examining the relationship between disease burden and socio-demographic development. We conducted frontier analysis to evaluate the potential for reducing the burden of CRC attributable to high BMI relative to SDI levels. This analysis identified the top 15 countries or regions with the largest deviations from the frontier, with effective differences (ef_df) ranging from 64.34 to 92.02. These countries include Hungary (92.02), Slovakia (82.14), Nauru (80.06), Bulgaria (79.87), Uruguay (78.33), Greenland (74.52), Croatia (74.38), Monaco (71.55), Barbados (70.30), Serbia (68.61), American Samoa (68.04), Poland (67.67), Romania (67.11), Republic of Moldova (67.07), and Czechia (64.34).

**Figure 8:**
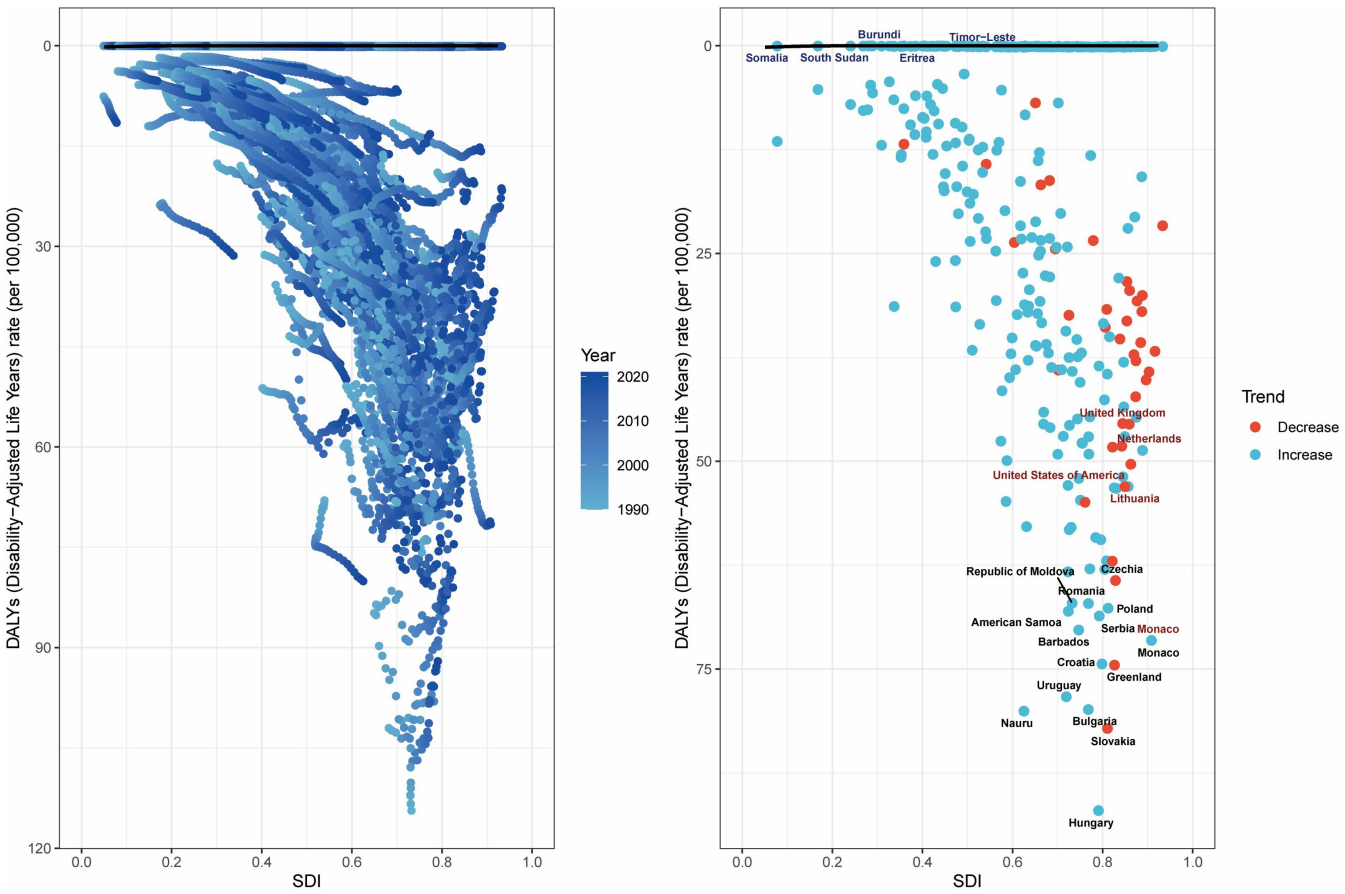
Frontier analysis of ASDR for CRC based on SDI in 2021.

Among low-SDI countries (<0.50), the five countries with the smallest deviations from the frontier were Timor-Leste (ef_df: 5.14), Burundi (ef_df: 5.68), South Sudan (ef_df: 7.69), Eritrea (ef_df: 8.74), and Somalia (ef_df: 11.49). Conversely, among high-SDI countries (>0.85), the five countries with the largest deviations from the frontier were the United Kingdom (ef_df: 45.53), the Netherlands (ef_df: 48.69), the United States of America (ef_df: 50.35), Lithuania (ef_df: 53.04), and Monaco (ef_df: 71.55) (Figure 8).

### 3.9 Projected burden of CRC attributable to high BMI: a BAPC modeling analysis

**Figure 9:**
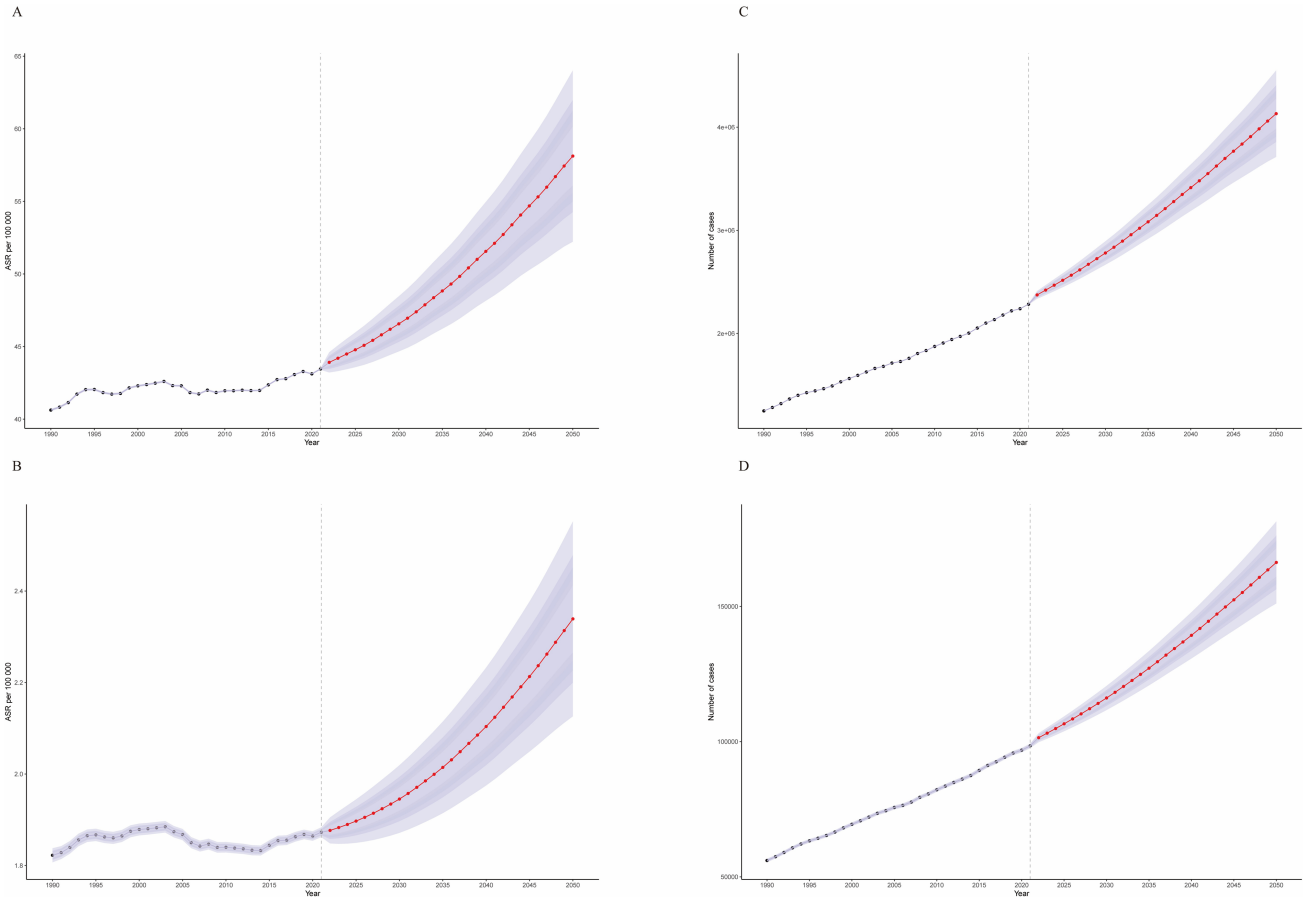
Temporal trends from 1990 to 2050. **(A)** Age-standardized disability-adjusted life year rates (ASDR). **(B)** Age-standardized mortality rates (ASMR). **(C)** Actual disability-adjusted life years (DALYs). **(D)** Actual number of deaths.

We conducted a BAPC analysis to project the future burden of CRC attributable to high BMI (Figure 9). Our projection analysis revealed a concerning upward trend in the global burden of high BMI-related CRC from 2022 to 2050. By 2050, the ASDR and ASMR for CRC attributable to high BMI are projected to increase to 58.12 (95% CI: 52.22, 64.02) per 100,000 population and 2.34 (95% CI: 2.13, 2.55), respectively. Detailed numerical projections are provided in Supplementary Tables S3, S4.

This projection translates to an estimated 166,256.88 (95% CI: 151,101.77, 181,411.99) deaths and a loss of 4,131,221.82 (95% CI: 3,711,846.43, 4,550,597.22) life years in 2050 alone. Furthermore, our age-stratified analysis demonstrated a significant increasing trend in the burden of high BMI-related CRC across all age groups from 2022 to 2050, with a more pronounced escalation observed in older age groups (Supplementary Figures S8–S11).

## 4 Discussion

Leveraging the authoritative GBD 2021 database and employing multidimensional analytical approaches, this study systematically elucidates the heterogeneous global burden of CRC attributable to high BMI. The standardized metrics from GBD, including ASMR and ASDR, ensure the validity of cross-national comparisons. The synergistic application of decomposition analysis, APC modeling, and frontier analysis not only quantifies the pivotal role of population growth as a core driver but also identifies performance disparities in high SDI regions, particularly Central Europe. These findings underscore the necessity of transcending unidimensional economic indicators when formulating region-specific prevention and control strategies.

Furthermore, our health inequality analysis reveals a non-linear relationship between SDI and disease burden, highlighting the complex trade-offs between economic development and chronic disease control. Our projections of future burden trends through 2050 aim to provide policymakers with critical insights and evidence-based guidance for efficient resource allocation and the development of targeted prevention strategies.

The analysis of temporal trends from 1990 to 2021 indicates that while the ASMR and ASDR attributable to high BMI showed no significant increase, with EAPCs of 0.12 and −0.0014 respectively, the absolute burden of CRC related to high BMI has substantially escalated. Both death counts and DALYs have more than doubled globally, with projections indicating a continued upward trajectory through 2050. This concerning trend is closely associated with the global surge in obesity prevalence and population aging (28, 29). From 1980 to 2013, the proportion of individuals with high BMI increased to over 30% in both males and females (30). East Asia (particularly China) and the United States bear the heaviest disease burden among 204 countries globally, which may be attributed to Western dietary patterns high in fat (31), sedentary lifestyles, and disparities in disease screening coverage (32, 33). Notably, although high-SDI countries (e.g., Hungary, Slovakia) exhibit the highest ASMR and ASDR, their EAPCs show a declining trend, likely due to well-established routine and early cancer screening systems and effective obesity prevention policies (34, 35). In contrast, the high EAPCs in low-SDI regions reflect delays in diagnosis caused by insufficient healthcare resources (17, 36). We acknowledge that region-specific public health interventions and policies may influence the observed geographic disparities in BMI-associated CRC burden. For instance, national CRC screening programs initiated in 2003 across European countries, such as the UK’s Fecal Occult Blood Test (FOBT) initiative, have demonstrated effectiveness in reducing CRC-related mortality rates. Similarly, Australia and Canada have stabilized population-level BMI trends through legislative restrictions on advertising of sugar-sweetened beverages and fast food, coupled with nationwide school-based nutrition education campaigns. In Latin America, Chile introduced a sugar-sweetened beverage tax in 2016 to curb rising obesity rates. Meanwhile, medium-SDI countries like China and Mexico implemented sugar-sweetened beverage taxation and national weight management guidelines between 2016 and 2020, though limited coverage of these measures has coincided with persistent increases in BMI-attributable CRC burden during the same period. These policy variations partially explain the divergent trajectories in BMI-attributable CRC burden growth rates across SDI regions. Nevertheless, we emphasize the methodological challenges in precisely quantifying these effects, particularly due to temporal lags between policy implementation, behavioral adaptation, and measurable health outcomes. Robust longitudinal evaluations incorporating policy effectiveness metrics will be essential to disentangle these complex causal relationships in future research.

The findings of this study align with previous GBD analyses, confirming high BMI as a significant modifiable risk factor for CRC (10), while further elucidating its regional heterogeneity (39, 40). For instance, the high burden in East Asia is consistent with the enhanced BMI-CRC risk association in Asian populations reported by Bardou et al. (13). Meanwhile, the exceptionally high ASMR in Central European countries (e.g., 3.79 per 100,000 in Hungary) may be linked to high red meat consumption, rising obesity prevalence, and alcohol consumption culture in the region (16). Additionally, decomposition analysis revealed that population growth is the primary driver of the increasing global burden (contributing 68%), consistent with the findings of Dai et al. (8). Regarding BMI-related disease burden. The non-linear relationship between the SDI and the burden of CRC (peaking at SDI = 0.75) highlights the complex trade-offs between economic development and health risks. Although high-SDI countries possess stronger healthcare capacities, the prevalence of obesity and population aging partially offset these advantages. This finding challenges the simplistic assumption that “higher economic development leads to better health outcomes.” Conversely, low-SDI regions, constrained by limited screening technologies and preventive awareness, may underestimate the actual disease burden despite lower detection rates (37). The declining trends in the SII and CI from 1990 to 2021 indicate a slight reduction in global health disparities. However, the persistent excess burden in high-SDI countries underscores the need for enhanced international collaboration to optimize resource allocation (18).

The study findings support the integration of BMI control into primary prevention strategies for CRC, with region-specific policies tailored to different SDI levels. For high-SDI regions (SDI > 0.81), optimizing screening systems to include BMI as a high-risk stratification indicator and strengthening obesity prevention measures, such as community-based exercise interventions (e.g., the “Let us Move!” initiative in the United States), are recommended. For middle-high and middle-SDI regions (SDI 0.61–0.81), efforts should focus on improving early CRC diagnosis capabilities, promoting low-cost screening tools, and enhancing health education. For middle-low and low SDI regions (SDI < 0.61), based on our findings, we recommend strengthening primary prevention education to promote healthy dietary habits and regular physical activity to curb the rising prevalence of overweight and obesity; implementing health education campaigns to raise public awareness of the link between elevated BMI and CRC risk; gradually introducing and expanding cost-effective CRC screening programs tailored to local resources and healthcare system capacities, particularly targeting high-risk populations; enhancing the capacity of healthcare systems in cancer prevention, early diagnosis, and treatment; and calling for the international community to provide technical and financial support to low-SDI countries to implement effective cancer control strategies.

Furthermore, the “performance deviation” observed in countries like Hungary and Slovakia in the frontier analysis—where the actual burden significantly exceeds SDI-based expectations—serves as a critical warning. It emphasizes the need for regionally precise prevention strategies rather than relying solely on economic development metrics. This situation highlights the urgent necessity for these high-SDI countries to optimize health policies more effectively.

This study has several limitations. First, the GBD data rely on modeled estimates, which may underestimate the true burden in low-resource settings. Additionally, variations in data quality, quantity, etiological assessments, and diagnostic accuracy across regions may introduce heterogeneity (38). Moreover, the GBD 2021 database lacks subtype-specific classification for colon cancer versus rectal cancer, which may obscure differences in burden patterns and the distinct effects of elevated BMI across these subtypes. This limitation underscores the need for refined data granularity to elucidate pathophysiological heterogeneity in future epidemiological studies. Second, our study focused exclusively on BMI and did not account for its potential interactions with other behavioral or environmental risk factors (e.g., diet, physical activity, smoking, diabetes). This simplified modeling approach may compromise the accuracy and robustness of the findings by overlooking synergistic or antagonistic effects between risk variables. Future investigations should prioritize integrating multidimensional risk factors into comprehensive analyses to better capture the complex etiology of colorectal cancer and refine risk attribution frameworks. Finally, the BAPC projections assume that current trends will remain unchanged, whereas actual interventions may alter these trajectories. Future research should focus on multicenter prospective cohort studies to explore the molecular mechanisms underlying the BMI-CRC relationship using genomic approaches and to evaluate the long-term effects of policy interventions, such as sugar taxes.

This study systematically elucidates the global heterogeneity in the burden of CRC attributable to high BMI, emphasizing the need for region-specific prevention and control strategies tailored to socioeconomic contexts.

## 5 Conclusion

The study reveals that from 1990 to 2021, the ASMR and ASDR attributable to high BMI showed no significant increase. However, both the absolute number of deaths and DALYs associated with high BMI demonstrated a robust upward trend, more than doubling since 1990. East Asia exhibited the highest mortality risk and DALY risk for colorectal cancer attributable to high BMI, with China and the United States bearing the heaviest burden. Significant disparities in colorectal cancer burden related to high BMI were observed across countries and regions with different SDI levels. These findings provide critical evidence for global health policymakers: optimizing obesity prevention strategies in high-SDI countries, enhancing early diagnostic capabilities in low-SDI regions, and fostering international collaboration to reduce health disparities. Such measures are essential to address the dual challenges of population aging and the obesity epidemic.

## Data Availability

Publicly available datasets were analyzed in this study. This data can be found: https://www.healthdata.org/research-analysis/gbd.

## Footnotes

^1^ http://ghdx.healthdata.org/gbd-results-tool
