# Correction: Analysis of the burden of colorectal cancer attributable to high body mass index in 204 countries and regions worldwide from 1990 to 2021

**DOI:** 10.3389/fnut.2026.1790081

**Published:** 2026-02-17

**Authors:** Mi Zhao, Ya Zheng, Zhaofeng Chen

**Affiliations:** 1 The First Clinical Medical College, Lanzhou University, Lanzhou, China; 2 Department of Gastroenterology, The First Hospital of Lanzhou University, Lanzhou, China; 3 Gansu Province Clinical Research Center for Digestive Diseases, The First Hospital of Lanzhou University, Lanzhou, China

**Keywords:** colorectal cancer, Global Burden of Disease, high body mass index, health inequality, socio-demographic Index

In the published article, two references were erroneously omitted from the **Discussion**, paragraph 4. The incorrect sentence reads as follows: “The findings of this study align with previous GBD analyses, confirming high BMI as a significant modifiable risk factor for CRC (**10**), while further elucidating its regional heterogeneity.” The correct sentence should read as, “The findings of this study align with previous GBD analyses, confirming high BMI as a significant modifiable risk factor for CRC (**10**), while further elucidating its regional heterogeneity (39, 40).”

The following references have been added to the reference list:

39. Zhang Q, Feng J, Xu Z, Guo Y, Zhu B, Qian P. Burden of colorectal cancer attributable to high body-mass index in 204 countries and territories, 1990-2021: results from the Global Burden of Disease Study 2021. *Public Health*. (2025) 242:388–98. doi: 10.1016/j.puhe.2025.02.040

40. Jin X, Dong D, Xu Z, Sun M. The global burden of colorectal cancer attributable to high body-mass index in 204 countries and territories: findings from 1990 to 2021 and predictions to 2035. *Front Nutr*. (2024) 11:1473851. doi: 10.3389/fnut.2024.1473851

The original version of this article has been updated.

