# Supplementary material for: Analysis of the burden of colorectal cancer attributable to high body mass index in 204 countries and regions worldwide from 1990 to 2021
Source: Front Nutr. 2025 Jun 9;12:1589250. doi: 10.3389/fnut.2025.1589250 (PMC12183065; doi:10.3389/fnut.2025.1589250)
Supplement: Supplementary file 1 [file Table_1.docx]

*Supplementary Material*

**Supplementary file 2:**Supplementary tables

#### **Table S1.**Deaths and ASMR of CRC attributable to the high BMI in 1990 and 2021 and the EAPC from 1990 to 2021.

| Deaths | 1990 |  | 2021 |  | EAPC(1990-2021) |  |
| --- | --- | --- | --- | --- | --- | --- |
| location | Death Cases(95%UI) | ASMR(95%UI) | Death Cases(95%UI) | ASMR(95%UI) | ASMR(95%CI) |  |
| Global | 41535.76(17665.60,67379.01) | 1.14(0.48,1.86) | 99267.99(42956.34,157948.81) | 1.17(0.51,1.87) | -0.00(-0.04,0.04) |  |
| Afghanistan | 58.02(14.00,113.63) | 0.81(0.21,1.57) | 116.66(37.55,221.52) | 1.11(0.38,2.07) | 1.12(1.01,1.24) |  |
| Albania | 15.21(6.28,25.46) | 0.81(0.33,1.37) | 42.36(17.89,71.35) | 0.97(0.41,1.63) | 0.87(0.66,1.08) |  |
| Algeria | 37.52(14.71,63.00) | 0.36(0.14,0.62) | 182.90(74.71,298.66) | 0.59(0.24,0.97) | 1.92(1.74,2.09) |  |
| American Samoa | 0.45(0.20,0.71) | 2.03(0.90,3.19) | 1.20(0.58,1.85) | 2.59(1.23,4.01) | 0.92(0.81,1.02) |  |
| Andorra | 1.18(0.44,2.20) | 2.13(0.79,3.95) | 2.64(1.04,4.66) | 1.65(0.65,2.91) | -0.49(-0.77,-0.21) |  |
| Angola | 8.27(2.97,14.55) | 0.21(0.08,0.36) | 54.33(19.87,97.91) | 0.46(0.17,0.83) | 2.55(2.41,2.69) |  |
| Antigua and Barbuda | 0.52(0.22,0.85) | 0.96(0.40,1.56) | 1.81(0.75,2.87) | 1.77(0.73,2.82) | 2.08(1.90,2.27) |  |
| Argentina | 699.53(303.83,1143.70) | 2.21(0.97,3.61) | 1532.47(671.25,2506.57) | 2.70(1.19,4.42) | 0.96(0.72,1.20) |  |
| Armenia | 40.54(16.90,64.06) | 1.50(0.62,2.39) | 72.92(31.18,117.30) | 1.67(0.71,2.69) | 0.69(0.51,0.88) |  |
| Australia | 440.63(182.99,698.37) | 2.27(0.95,3.61) | 916.34(395.19,1454.00) | 1.94(0.84,3.09) | -0.75(-0.84,-0.65) |  |
| Austria | 276.31(110.04,448.26) | 2.27(0.91,3.67) | 255.23(107.28,437.65) | 1.30(0.54,2.20) | -1.86(-1.92,-1.80) |  |
| Azerbaijan | 46.68(18.60,75.59) | 0.92(0.37,1.49) | 96.14(41.27,159.92) | 0.93(0.40,1.57) | 0.40(0.15,0.66) |  |
| Bahamas | 2.30(0.97,3.71) | 1.49(0.62,2.39) | 9.79(3.99,15.89) | 2.47(1.01,4.02) | 1.93(1.80,2.07) |  |
| Bahrain | 1.97(0.82,3.19) | 1.24(0.52,2.03) | 12.49(5.31,20.28) | 1.70(0.70,2.76) | 0.61(0.32,0.91) |  |
| Bangladesh | 28.47(8.04,49.21) | 0.06(0.02,0.10) | 164.76(57.40,291.12) | 0.12(0.04,0.21) | 2.55(2.41,2.70) |  |
| Barbados | 5.05(2.07,8.32) | 1.72(0.71,2.85) | 15.91(6.88,26.62) | 3.05(1.32,5.11) | 2.19(1.90,2.48) |  |
| Belarus | 215.99(87.52,356.77) | 1.66(0.67,2.74) | 395.13(163.62,669.86) | 2.42(1.00,4.11) | 0.53(0.20,0.85) |  |
| Belgium | 306.70(128.21,493.40) | 1.96(0.81,3.14) | 387.53(163.81,651.96) | 1.51(0.64,2.54) | -0.77(-0.88,-0.66) |  |
| Belize | 0.61(0.26,1.00) | 0.66(0.28,1.08) | 3.78(1.67,5.92) | 1.30(0.57,2.05) | 2.23(1.71,2.75) |  |
| Benin | 4.58(1.72,7.52) | 0.23(0.09,0.39) | 19.21(7.39,32.66) | 0.39(0.14,0.66) | 1.82(1.72,1.91) |  |
| Bermuda | 1.78(0.73,2.97) | 2.95(1.21,4.91) | 3.95(1.72,6.42) | 2.74(1.20,4.46) | -0.11(-0.28,0.05) |  |
| Bhutan | 0.64(0.22,1.17) | 0.24(0.08,0.44) | 2.09(0.80,3.83) | 0.34(0.13,0.62) | 1.12(1.03,1.21) |  |
| Bolivia (Plurinational State of) | 28.51(10.15,52.82) | 0.91(0.33,1.70) | 129.23(50.52,226.04) | 1.48(0.57,2.60) | 1.59(1.56,1.62) |  |
| Bosnia and Herzegovina | 51.35(20.85,84.44) | 1.29(0.52,2.12) | 145.08(62.65,237.72) | 2.27(0.98,3.71) | 2.15(1.93,2.38) |  |
| Botswana | 2.57(0.98,4.68) | 0.51(0.20,0.91) | 13.28(5.31,22.97) | 1.03(0.41,1.75) | 2.64(2.34,2.93) |  |
| Brazil | 687.44(284.49,1114.92) | 0.81(0.33,1.30) | 3555.35(1501.85,5722.52) | 1.42(0.60,2.29) | 1.84(1.72,1.95) |  |
| Brunei Darussalam | 1.20(0.49,2.07) | 1.09(0.42,1.87) | 5.27(2.14,8.75) | 1.39(0.55,2.33) | 1.33(1.09,1.58) |  |
| Bulgaria | 283.51(124.54,461.39) | 2.37(1.03,3.85) | 488.76(203.93,818.80) | 3.36(1.41,5.62) | 1.56(1.36,1.76) |  |
| Burkina Faso | 4.51(1.46,7.48) | 0.11(0.03,0.18) | 16.83(5.49,29.71) | 0.19(0.06,0.33) | 2.05(1.90,2.20) |  |
| Burundi | 4.11(1.25,7.28) | 0.18(0.05,0.32) | 10.63(3.44,18.91) | 0.23(0.07,0.40) | 0.37(0.14,0.59) |  |
| Cabo Verde | 0.37(0.14,0.63) | 0.16(0.06,0.27) | 3.10(1.24,5.15) | 0.73(0.29,1.21) | 4.42(4.03,4.81) |  |
| Cambodia | 11.10(3.18,19.73) | 0.23(0.07,0.42) | 54.04(18.55,91.47) | 0.42(0.14,0.70) | 1.93(1.82,2.03) |  |
| Cameroon | 20.00(7.96,33.15) | 0.48(0.19,0.81) | 98.65(35.61,171.78) | 0.87(0.32,1.51) | 2.09(2.01,2.18) |  |
| Canada | 705.26(308.02,1129.16) | 2.17(0.95,3.47) | 1365.42(602.27,2185.14) | 1.82(0.80,2.90) | -0.43(-0.55,-0.30) |  |
| Central African Republic | 2.67(0.93,4.90) | 0.23(0.08,0.40) | 10.13(3.41,18.76) | 0.43(0.15,0.79) | 2.17(2.13,2.21) |  |
| Chad | 3.98(1.47,6.60) | 0.15(0.05,0.24) | 15.77(6.24,26.65) | 0.29(0.12,0.49) | 2.45(2.35,2.55) |  |
| Chile | 131.98(54.79,213.82) | 1.38(0.57,2.23) | 501.01(226.90,810.03) | 1.93(0.88,3.12) | 1.39(1.27,1.50) |  |
| China | 3611.38(1251.93,6159.88) | 0.45(0.15,0.77) | 19417.57(8052.91,32452.37) | 0.94(0.39,1.58) | 2.39(2.31,2.48) |  |
| Colombia | 123.88(50.58,200.57) | 0.74(0.30,1.19) | 705.33(298.95,1175.49) | 1.28(0.54,2.13) | 1.64(1.49,1.79) |  |
| Comoros | 0.59(0.19,1.04) | 0.31(0.10,0.54) | 3.23(1.16,5.78) | 0.69(0.25,1.22) | 2.58(2.46,2.70) |  |
| Congo | 4.54(1.65,8.14) | 0.41(0.15,0.74) | 20.54(7.65,35.54) | 0.74(0.28,1.26) | 1.72(1.60,1.83) |  |
| Cook Islands | 0.12(0.05,0.20) | 1.02(0.44,1.65) | 0.24(0.11,0.37) | 0.92(0.43,1.47) | -0.46(-0.63,-0.29) |  |
| Costa Rica | 14.80(6.08,24.22) | 0.86(0.35,1.42) | 106.05(44.69,173.16) | 1.92(0.81,3.15) | 2.88(2.68,3.08) |  |
| Cote d'Ivoire | 7.80(3.06,13.03) | 0.21(0.08,0.34) | 33.77(12.64,58.64) | 0.32(0.12,0.55) | 1.44(1.34,1.55) |  |
| Croatia | 146.90(62.77,238.48) | 2.55(1.09,4.10) | 316.48(137.75,515.02) | 3.36(1.46,5.44) | 1.04(0.84,1.24) |  |
| Cuba | 104.94(42.75,166.49) | 1.04(0.42,1.65) | 375.92(152.70,619.17) | 1.89(0.77,3.11) | 2.12(2.02,2.22) |  |
| Cyprus | 9.05(3.45,14.68) | 1.32(0.50,2.12) | 26.79(11.31,45.20) | 1.37(0.58,2.30) | 0.44(0.29,0.59) |  |
| Czechia | 616.27(273.08,976.26) | 4.40(1.95,7.00) | 641.23(282.67,1038.27) | 2.86(1.26,4.65) | -1.71(-1.90,-1.51) |  |
| Democratic People's Republic of Korea | 40.15(11.80,74.74) | 0.28(0.09,0.51) | 162.61(56.80,303.72) | 0.52(0.18,0.97) | 2.21(2.11,2.32) |  |
| Democratic Republic of the Congo | 29.27(9.71,50.75) | 0.20(0.07,0.35) | 148.92(52.68,268.57) | 0.44(0.15,0.80) | 2.66(2.42,2.90) |  |
| Denmark | 155.77(65.41,253.84) | 1.88(0.79,3.07) | 242.48(102.85,413.01) | 1.90(0.81,3.24) | -0.32(-0.61,-0.03) |  |
| Djibouti | 0.24(0.07,0.47) | 0.18(0.05,0.34) | 2.40(0.78,4.28) | 0.39(0.12,0.71) | 2.56(2.52,2.61) |  |
| Dominica | 0.86(0.36,1.37) | 1.45(0.61,2.33) | 1.88(0.84,2.96) | 2.31(1.03,3.68) | 1.62(1.54,1.70) |  |
| Dominican Republic | 15.63(6.03,26.54) | 0.44(0.17,0.73) | 88.80(35.55,148.82) | 0.89(0.36,1.49) | 2.77(2.58,2.97) |  |
| Ecuador | 29.80(12.20,47.85) | 0.59(0.24,0.94) | 202.71(84.40,331.81) | 1.26(0.52,2.07) | 2.91(2.57,3.25) |  |
| Egypt | 168.38(72.17,265.97) | 0.64(0.27,1.02) | 926.52(416.11,1463.04) | 1.58(0.71,2.49) | 3.70(3.39,4.01) |  |
| El Salvador | 15.47(6.35,24.72) | 0.52(0.21,0.83) | 72.25(31.20,120.05) | 1.15(0.50,1.92) | 2.62(2.43,2.81) |  |
| Equatorial Guinea | 0.75(0.29,1.33) | 0.38(0.15,0.68) | 4.97(1.94,9.25) | 1.03(0.40,1.90) | 3.47(3.31,3.62) |  |
| Eritrea | 1.80(0.63,3.13) | 0.15(0.05,0.27) | 9.16(3.30,15.52) | 0.34(0.12,0.58) | 2.64(2.58,2.69) |  |
| Estonia | 42.97(17.76,69.37) | 2.08(0.86,3.35) | 68.86(30.23,114.31) | 2.36(1.03,3.90) | 0.14(-0.02,0.30) |  |
| Eswatini | 2.81(1.13,4.84) | 1.13(0.44,1.98) | 11.59(4.64,20.24) | 2.31(0.95,3.95) | 2.73(2.18,3.28) |  |
| Ethiopia | 87.70(26.95,149.67) | 0.45(0.14,0.75) | 199.50(70.54,329.74) | 0.48(0.17,0.81) | -0.15(-0.39,0.09) |  |
| Fiji | 3.29(1.36,5.55) | 0.95(0.38,1.61) | 10.45(4.49,17.00) | 1.48(0.63,2.39) | 1.46(1.28,1.64) |  |
| Finland | 99.66(41.22,162.91) | 1.37(0.57,2.26) | 184.38(78.89,307.88) | 1.34(0.57,2.23) | -0.14(-0.23,-0.04) |  |
| France | 1518.49(624.48,2486.45) | 1.77(0.72,2.89) | 2573.87(1077.83,4254.29) | 1.61(0.68,2.65) | -0.28(-0.38,-0.17) |  |
| Gabon | 4.92(1.72,9.45) | 0.87(0.30,1.66) | 15.23(6.22,26.76) | 1.51(0.62,2.63) | 1.72(1.63,1.82) |  |
| Gambia | 0.48(0.17,0.83) | 0.14(0.05,0.25) | 2.27(0.88,3.98) | 0.24(0.09,0.41) | 1.58(1.42,1.73) |  |
| Georgia | 64.94(26.24,102.38) | 1.03(0.42,1.63) | 91.16(39.74,147.92) | 1.53(0.67,2.50) | 2.48(2.00,2.96) |  |
| Germany | 3573.77(1485.21,5720.44) | 2.73(1.13,4.37) | 3630.45(1500.29,6089.46) | 1.75(0.72,2.93) | -1.74(-1.86,-1.62) |  |
| Ghana | 11.95(4.57,20.19) | 0.20(0.07,0.33) | 81.48(31.37,138.77) | 0.54(0.20,0.92) | 3.45(3.40,3.51) |  |
| Greece | 207.63(85.15,340.52) | 1.37(0.56,2.26) | 467.46(188.53,770.40) | 1.75(0.72,2.88) | 0.46(0.24,0.68) |  |
| Greenland | 1.46(0.63,2.41) | 4.73(2.05,7.77) | 2.03(0.85,3.38) | 3.10(1.29,5.11) | -1.40(-1.50,-1.30) |  |
| Grenada | 0.66(0.26,1.07) | 0.94(0.37,1.51) | 1.98(0.83,3.23) | 1.80(0.75,2.95) | 2.46(2.31,2.61) |  |
| Guam | 1.17(0.48,1.91) | 1.66(0.69,2.75) | 2.98(1.31,4.77) | 1.42(0.63,2.27) | 0.05(-0.31,0.42) |  |
| Guatemala | 13.78(5.84,22.32) | 0.44(0.19,0.72) | 95.01(41.07,156.02) | 0.88(0.38,1.44) | 2.10(1.79,2.41) |  |
| Guinea | 4.98(1.82,8.39) | 0.15(0.06,0.26) | 14.09(5.10,23.89) | 0.26(0.09,0.43) | 1.78(1.74,1.81) |  |
| Guinea-Bissau | 1.08(0.38,1.92) | 0.27(0.10,0.49) | 3.58(1.42,6.11) | 0.52(0.21,0.86) | 2.23(2.16,2.30) |  |
| Guyana | 3.03(1.17,4.97) | 0.82(0.31,1.34) | 8.73(3.82,14.68) | 1.38(0.60,2.34) | 2.05(1.79,2.32) |  |
| Haiti | 12.25(3.86,21.74) | 0.38(0.12,0.67) | 47.56(18.16,84.78) | 0.66(0.25,1.20) | 2.07(1.96,2.18) |  |
| Honduras | 6.99(2.69,11.65) | 0.34(0.13,0.57) | 44.67(18.80,75.79) | 0.73(0.31,1.23) | 2.59(2.39,2.78) |  |
| Hungary | 532.18(220.30,824.02) | 3.63(1.52,5.62) | 753.60(341.42,1229.80) | 3.79(1.72,6.19) | 0.05(-0.19,0.30) |  |
| Iceland | 4.78(2.03,7.70) | 1.64(0.70,2.64) | 8.53(3.60,14.43) | 1.39(0.58,2.33) | -0.45(-0.59,-0.31) |  |
| India | 387.23(132.08,618.99) | 0.08(0.03,0.13) | 2317.48(914.21,3580.26) | 0.19(0.08,0.30) | 2.92(2.84,3.00) |  |
| Indonesia | 186.55(57.93,318.19) | 0.18(0.05,0.30) | 1235.51(494.20,2132.79) | 0.50(0.20,0.86) | 3.56(3.37,3.74) |  |
| Iran (Islamic Republic of) | 126.53(53.93,205.84) | 0.51(0.22,0.82) | 742.51(319.23,1178.64) | 0.99(0.43,1.58) | 2.61(2.39,2.84) |  |
| Iraq | 50.34(20.83,81.84) | 0.63(0.25,1.02) | 213.86(90.29,356.96) | 0.94(0.39,1.56) | 1.36(1.14,1.57) |  |
| Ireland | 91.08(37.00,150.92) | 2.26(0.92,3.74) | 139.19(59.46,235.03) | 1.70(0.73,2.88) | -0.75(-0.85,-0.65) |  |
| Israel | 98.62(41.35,154.86) | 2.07(0.87,3.28) | 199.62(87.19,327.81) | 1.52(0.67,2.51) | -1.60(-1.93,-1.26) |  |
| Italy | 1457.07(597.90,2382.65) | 1.62(0.66,2.64) | 2543.07(1082.66,4139.22) | 1.56(0.66,2.56) | -0.22(-0.38,-0.07) |  |
| Jamaica | 15.21(6.39,24.16) | 0.84(0.36,1.34) | 57.50(25.36,96.21) | 1.84(0.81,3.06) | 2.62(2.27,2.96) |  |
| Japan | 1276.52(470.09,2060.40) | 0.76(0.28,1.23) | 3499.63(1339.75,5561.59) | 0.87(0.34,1.39) | 0.30(0.23,0.37) |  |
| Jordan | 18.04(7.67,29.65) | 1.37(0.58,2.26) | 108.46(47.17,175.08) | 1.57(0.69,2.54) | 0.47(0.23,0.71) |  |
| Kazakhstan | 189.60(78.21,310.36) | 1.52(0.62,2.49) | 226.80(98.97,371.67) | 1.30(0.57,2.11) | -0.21(-0.47,0.05) |  |
| Kenya | 14.51(5.27,23.73) | 0.17(0.06,0.28) | 109.85(42.41,183.88) | 0.48(0.19,0.79) | 3.84(3.62,4.06) |  |
| Kiribati | 0.33(0.13,0.54) | 0.84(0.32,1.40) | 0.89(0.37,1.43) | 1.21(0.49,1.93) | 1.04(0.96,1.13) |  |
| Kuwait | 4.21(1.82,6.65) | 0.74(0.32,1.20) | 45.20(21.25,72.82) | 1.66(0.77,2.69) | 3.07(2.57,3.57) |  |
| Kyrgyzstan | 33.51(13.48,54.43) | 1.13(0.46,1.82) | 42.89(17.68,67.36) | 0.91(0.37,1.43) | -0.51(-0.68,-0.34) |  |
| Lao People's Democratic Republic | 5.97(1.78,11.47) | 0.28(0.09,0.54) | 25.16(9.23,45.17) | 0.53(0.20,0.95) | 2.12(2.03,2.22) |  |
| Latvia | 76.06(32.02,123.90) | 2.11(0.89,3.43) | 97.55(42.28,160.69) | 2.32(1.01,3.83) | 0.40(0.21,0.60) |  |
| Lebanon | 28.96(11.04,50.83) | 1.42(0.54,2.50) | 106.09(44.32,171.48) | 1.69(0.71,2.72) | 0.99(0.77,1.21) |  |
| Lesotho | 4.04(1.53,6.78) | 0.51(0.19,0.86) | 15.05(5.71,26.62) | 1.49(0.56,2.63) | 4.48(3.94,5.02) |  |
| Liberia | 3.59(1.32,5.77) | 0.32(0.12,0.51) | 11.02(3.69,20.39) | 0.54(0.18,0.99) | 2.02(1.79,2.26) |  |
| Libya | 23.81(10.02,40.55) | 1.30(0.53,2.22) | 119.34(47.84,201.00) | 2.36(0.93,4.00) | 2.33(2.15,2.51) |  |
| Lithuania | 79.71(34.37,129.13) | 1.77(0.76,2.85) | 143.92(64.36,237.79) | 2.35(1.04,3.85) | 0.92(0.74,1.10) |  |
| Luxembourg | 13.04(5.39,20.82) | 2.40(0.99,3.83) | 19.38(8.50,31.21) | 1.71(0.75,2.75) | -1.06(-1.25,-0.86) |  |
| Madagascar | 9.42(3.26,16.11) | 0.19(0.07,0.33) | 36.65(14.29,64.03) | 0.35(0.14,0.62) | 2.00(1.83,2.17) |  |
| Malawi | 4.08(1.32,6.97) | 0.11(0.04,0.19) | 17.00(6.05,29.97) | 0.24(0.09,0.41) | 2.25(2.08,2.42) |  |
| Malaysia | 74.64(29.81,118.03) | 0.79(0.32,1.25) | 385.78(161.11,617.31) | 1.39(0.58,2.23) | 1.66(1.53,1.78) |  |
| Maldives | 0.25(0.08,0.44) | 0.24(0.08,0.42) | 0.91(0.36,1.52) | 0.25(0.10,0.42) | -0.17(-0.30,-0.04) |  |
| Mali | 8.75(3.12,14.38) | 0.23(0.08,0.37) | 26.27(9.40,45.50) | 0.31(0.11,0.53) | 1.12(0.98,1.27) |  |
| Malta | 5.81(2.30,9.35) | 1.39(0.55,2.23) | 15.55(6.61,26.18) | 1.50(0.65,2.54) | 0.18(0.05,0.31) |  |
| Marshall Islands | 0.21(0.09,0.36) | 1.31(0.57,2.22) | 0.61(0.26,1.02) | 1.76(0.74,2.91) | 0.93(0.86,0.99) |  |
| Mauritania | 4.63(1.87,7.80) | 0.50(0.20,0.85) | 15.68(5.90,26.68) | 0.81(0.30,1.37) | 1.58(1.42,1.74) |  |
| Mauritius | 3.32(1.35,5.43) | 0.46(0.19,0.74) | 23.54(9.86,37.55) | 1.29(0.54,2.04) | 2.85(2.66,3.05) |  |
| Mexico | 248.67(105.46,400.44) | 0.62(0.26,0.99) | 1582.34(698.34,2518.77) | 1.25(0.55,2.00) | 2.39(2.25,2.53) |  |
| Micronesia (Federated States of) | 0.69(0.28,1.20) | 1.40(0.57,2.45) | 1.36(0.55,2.28) | 1.84(0.72,3.02) | 0.87(0.83,0.90) |  |
| Monaco | 1.88(0.74,3.15) | 2.55(0.99,4.22) | 3.36(1.46,5.72) | 3.19(1.41,5.43) | 0.83(0.72,0.94) |  |
| Mongolia | 7.08(2.93,11.73) | 0.67(0.28,1.11) | 18.81(7.57,31.78) | 0.83(0.33,1.38) | 0.52(0.38,0.66) |  |
| Montenegro | 10.61(4.56,17.00) | 1.73(0.75,2.77) | 26.12(12.30,42.93) | 2.72(1.28,4.47) | 1.62(1.50,1.73) |  |
| Morocco | 64.34(22.78,108.74) | 0.46(0.16,0.77) | 325.98(124.21,578.47) | 0.97(0.37,1.69) | 2.69(2.58,2.80) |  |
| Mozambique | 4.19(1.36,7.26) | 0.08(0.03,0.14) | 18.31(6.67,31.82) | 0.18(0.06,0.32) | 3.25(3.06,3.44) |  |
| Myanmar | 68.92(22.70,134.12) | 0.28(0.09,0.53) | 218.08(79.73,380.28) | 0.43(0.16,0.76) | 1.27(1.16,1.37) |  |
| Namibia | 1.88(0.73,3.04) | 0.31(0.12,0.50) | 8.65(3.54,14.52) | 0.67(0.28,1.11) | 2.46(2.18,2.74) |  |
| Nauru | 0.12(0.04,0.21) | 2.30(0.86,4.15) | 0.17(0.07,0.29) | 2.82(1.07,4.89) | 0.58(0.54,0.62) |  |
| Nepal | 7.55(2.17,13.82) | 0.07(0.02,0.13) | 37.73(13.21,65.65) | 0.15(0.05,0.27) | 2.79(2.37,3.21) |  |
| Netherlands | 405.81(170.59,673.11) | 2.00(0.84,3.31) | 782.59(329.63,1281.14) | 2.12(0.90,3.48) | 0.30(0.12,0.48) |  |
| New Zealand | 119.92(50.14,195.19) | 3.09(1.29,5.01) | 207.09(85.16,338.83) | 2.37(0.97,3.86) | -0.89(-0.97,-0.81) |  |
| Nicaragua | 7.00(2.88,11.35) | 0.46(0.19,0.76) | 39.32(16.91,63.68) | 0.82(0.35,1.33) | 2.29(2.00,2.58) |  |
| Niger | 3.96(1.37,6.63) | 0.15(0.05,0.25) | 16.55(6.04,29.48) | 0.22(0.08,0.40) | 1.31(1.22,1.40) |  |
| Nigeria | 99.44(40.08,166.78) | 0.24(0.10,0.41) | 414.77(167.03,676.18) | 0.51(0.21,0.85) | 2.59(2.51,2.66) |  |
| Niue | 0.03(0.01,0.05) | 1.22(0.52,2.06) | 0.04(0.02,0.06) | 1.78(0.80,2.83) | 1.11(1.06,1.16) |  |
| North Macedonia | 31.06(12.98,49.53) | 1.72(0.72,2.75) | 78.68(35.04,132.73) | 2.53(1.13,4.24) | 1.30(0.95,1.66) |  |
| Northern Mariana Islands | 0.34(0.15,0.57) | 1.89(0.82,3.12) | 1.23(0.57,1.95) | 2.50(1.14,3.95) | 0.92(0.71,1.14) |  |
| Norway | 138.10(57.20,222.95) | 1.96(0.81,3.17) | 187.28(81.44,306.48) | 1.73(0.75,2.83) | -0.55(-0.66,-0.45) |  |
| Oman | 2.10(0.80,3.62) | 0.31(0.12,0.53) | 10.25(4.09,16.06) | 0.56(0.23,0.89) | 2.47(2.21,2.73) |  |
| Pakistan | 93.01(31.56,155.17) | 0.17(0.06,0.28) | 520.64(209.88,857.80) | 0.42(0.17,0.70) | 3.12(2.86,3.38) |  |
| Palau | 0.17(0.07,0.28) | 1.80(0.71,3.05) | 0.41(0.18,0.65) | 2.07(0.87,3.39) | 0.59(0.50,0.67) |  |
| Palestine | 17.26(7.25,29.60) | 2.10(0.88,3.57) | 57.29(23.35,89.86) | 2.49(1.02,3.91) | 0.63(0.47,0.79) |  |
| Panama | 13.05(5.34,21.13) | 0.92(0.38,1.49) | 73.83(32.58,122.06) | 1.66(0.73,2.75) | 2.18(2.09,2.28) |  |
| Papua New Guinea | 3.93(1.46,7.56) | 0.20(0.07,0.38) | 13.65(5.23,23.56) | 0.23(0.09,0.41) | 0.48(0.42,0.54) |  |
| Paraguay | 11.76(4.85,19.65) | 0.54(0.23,0.91) | 74.42(31.92,126.25) | 1.31(0.56,2.23) | 3.22(3.03,3.41) |  |
| Peru | 76.11(30.25,128.59) | 0.65(0.26,1.09) | 340.41(145.89,593.22) | 1.02(0.44,1.78) | 1.36(1.17,1.55) |  |
| Philippines | 104.88(37.50,167.22) | 0.34(0.12,0.54) | 640.10(258.21,1031.58) | 0.76(0.31,1.22) | 2.82(2.67,2.97) |  |
| Poland | 1006.58(431.72,1646.35) | 2.32(1.00,3.79) | 2345.90(992.04,3845.92) | 3.13(1.32,5.13) | 0.76(0.57,0.95) |  |
| Portugal | 250.43(102.03,415.54) | 1.86(0.76,3.08) | 522.94(228.68,861.88) | 1.97(0.87,3.24) | 0.23(-0.02,0.47) |  |
| Puerto Rico | 56.70(23.88,90.81) | 1.60(0.67,2.58) | 143.41(65.41,232.01) | 2.01(0.92,3.19) | 0.67(0.43,0.91) |  |
| Qatar | 1.62(0.70,2.63) | 1.72(0.75,2.82) | 15.11(6.70,25.12) | 2.07(0.90,3.42) | 0.80(0.12,1.48) |  |
| Republic of Korea | 130.23(42.83,218.69) | 0.47(0.15,0.78) | 656.51(255.35,1067.45) | 0.70(0.27,1.14) | 1.20(1.01,1.39) |  |
| Republic of Moldova | 87.17(38.81,140.23) | 2.00(0.89,3.21) | 161.77(69.47,256.30) | 2.67(1.15,4.24) | 1.40(0.95,1.85) |  |
| Romania | 359.57(152.04,574.58) | 1.29(0.54,2.09) | 1048.76(425.83,1765.82) | 2.77(1.13,4.65) | 2.20(1.96,2.44) |  |
| Russian Federation | 3313.16(1434.26,5236.54) | 1.84(0.79,2.90) | 6486.09(2776.95,10321.13) | 2.68(1.15,4.26) | 1.05(0.88,1.22) |  |
| Rwanda | 7.73(2.39,13.77) | 0.27(0.08,0.48) | 22.93(7.34,40.69) | 0.38(0.12,0.67) | 0.32(-0.03,0.67) |  |
| Saint Kitts and Nevis | 0.51(0.20,0.83) | 1.37(0.54,2.24) | 1.34(0.58,2.16) | 2.09(0.89,3.36) | 1.90(1.73,2.07) |  |
| Saint Lucia | 0.68(0.27,1.11) | 0.82(0.32,1.35) | 2.67(1.14,4.35) | 1.12(0.48,1.83) | 0.67(0.46,0.89) |  |
| Saint Vincent and the Grenadines | 0.45(0.18,0.74) | 0.65(0.26,1.07) | 1.64(0.68,2.71) | 1.19(0.49,1.96) | 1.97(1.81,2.13) |  |
| Samoa | 1.01(0.43,1.59) | 1.19(0.51,1.90) | 2.16(0.98,3.53) | 1.51(0.67,2.46) | 0.75(0.69,0.81) |  |
| San Marino | 0.85(0.34,1.40) | 2.32(0.92,3.85) | 1.14(0.45,2.06) | 1.36(0.53,2.44) | -0.81(-1.17,-0.45) |  |
| Sao Tome and Principe | 0.23(0.09,0.38) | 0.37(0.14,0.62) | 0.84(0.34,1.36) | 0.85(0.33,1.41) | 2.79(2.73,2.86) |  |
| Saudi Arabia | 35.42(14.31,60.89) | 0.60(0.25,1.04) | 271.15(119.22,431.76) | 1.36(0.61,2.15) | 2.84(2.42,3.25) |  |
| Senegal | 7.96(2.93,13.57) | 0.26(0.09,0.44) | 33.78(12.27,57.24) | 0.47(0.17,0.78) | 2.08(1.91,2.25) |  |
| Serbia | 249.42(110.35,417.95) | 2.43(1.06,4.07) | 500.54(231.36,811.25) | 2.95(1.37,4.78) | 0.52(0.41,0.64) |  |
| Seychelles | 0.62(0.24,0.99) | 1.10(0.42,1.75) | 2.57(1.12,4.06) | 2.23(0.96,3.53) | 2.44(2.16,2.73) |  |
| Sierra Leone | 3.18(1.01,5.62) | 0.16(0.05,0.29) | 11.25(3.90,19.94) | 0.32(0.11,0.56) | 2.48(2.35,2.61) |  |
| Singapore | 16.98(6.03,28.35) | 0.77(0.27,1.29) | 78.58(32.31,128.12) | 0.92(0.38,1.51) | 0.35(0.14,0.57) |  |
| Slovakia | 204.43(90.26,335.89) | 3.40(1.50,5.56) | 342.52(156.18,548.62) | 3.53(1.61,5.66) | 0.10(0.00,0.19) |  |
| Slovenia | 62.78(26.72,101.06) | 2.52(1.07,4.06) | 109.58(47.07,181.85) | 2.28(0.98,3.77) | -0.51(-0.81,-0.21) |  |
| Solomon Islands | 0.88(0.32,1.59) | 0.62(0.22,1.10) | 3.32(1.22,5.63) | 0.91(0.34,1.53) | 1.26(1.11,1.40) |  |
| Somalia | 7.04(2.14,13.24) | 0.26(0.08,0.50) | 26.84(9.29,49.40) | 0.41(0.13,0.76) | 1.56(1.51,1.62) |  |
| South Africa | 168.79(71.97,269.48) | 0.85(0.36,1.37) | 676.69(288.19,1060.87) | 1.55(0.66,2.44) | 2.04(1.79,2.30) |  |
| South Sudan | 4.24(1.19,8.57) | 0.17(0.05,0.34) | 11.44(3.59,20.52) | 0.30(0.09,0.53) | 1.87(1.66,2.09) |  |
| Spain | 1081.97(457.48,1791.35) | 1.98(0.84,3.27) | 2371.94(1012.05,3948.70) | 2.21(0.96,3.63) | 0.36(0.17,0.54) |  |
| Sri Lanka | 16.78(6.22,26.96) | 0.16(0.06,0.26) | 72.05(29.36,130.63) | 0.27(0.11,0.49) | 2.05(1.86,2.24) |  |
| Sudan | 45.54(17.49,82.17) | 0.49(0.19,0.87) | 174.45(69.96,309.87) | 0.88(0.36,1.55) | 2.00(1.90,2.10) |  |
| Suriname | 1.83(0.69,2.99) | 0.73(0.27,1.18) | 7.81(3.25,13.14) | 1.24(0.51,2.09) | 2.06(1.84,2.27) |  |
| Sweden | 255.22(104.74,428.37) | 1.63(0.67,2.71) | 358.75(152.16,584.38) | 1.51(0.64,2.46) | -0.17(-0.34,-0.01) |  |
| Switzerland | 135.03(56.68,217.95) | 1.25(0.53,2.02) | 202.83(83.01,334.61) | 1.01(0.42,1.67) | -0.77(-0.92,-0.62) |  |
| Syrian Arab Republic | 35.23(14.21,57.51) | 0.68(0.27,1.12) | 137.86(55.42,229.47) | 1.13(0.45,1.89) | 1.53(1.36,1.70) |  |
| Taiwan (Province of China) | 121.51(46.70,195.08) | 0.79(0.30,1.27) | 790.61(330.07,1267.73) | 1.86(0.77,2.97) | 2.44(2.09,2.79) |  |
| Tajikistan | 19.95(8.19,31.92) | 0.73(0.30,1.17) | 31.25(12.79,53.56) | 0.54(0.22,0.92) | -0.97(-1.18,-0.77) |  |
| Thailand | 143.64(51.10,238.44) | 0.40(0.14,0.67) | 1080.91(425.39,1833.78) | 1.00(0.39,1.70) | 2.82(2.65,2.98) |  |
| Timor-Leste | 0.25(0.06,0.47) | 0.09(0.02,0.16) | 1.58(0.51,2.78) | 0.18(0.06,0.32) | 2.67(2.36,2.98) |  |
| Togo | 2.31(0.84,3.84) | 0.20(0.07,0.33) | 15.12(5.21,26.59) | 0.44(0.15,0.77) | 2.79(2.71,2.87) |  |
| Tokelau | 0.02(0.01,0.03) | 1.13(0.49,1.93) | 0.02(0.01,0.03) | 1.47(0.66,2.35) | 0.82(0.81,0.84) |  |
| Tonga | 0.50(0.22,0.78) | 0.91(0.39,1.46) | 0.98(0.45,1.55) | 1.23(0.57,1.95) | 0.97(0.85,1.09) |  |
| Trinidad and Tobago | 10.62(4.58,16.72) | 1.34(0.57,2.10) | 35.03(14.58,57.43) | 1.81(0.75,2.98) | 0.95(0.82,1.08) |  |
| Tunisia | 26.73(11.11,45.28) | 0.56(0.23,0.95) | 124.17(52.12,216.41) | 0.96(0.40,1.67) | 1.62(1.55,1.69) |  |
| Turkey | 571.81(233.59,931.78) | 1.70(0.69,2.78) | 1795.06(762.69,2896.50) | 1.97(0.83,3.18) | 0.40(0.03,0.78) |  |
| Turkmenistan | 12.40(5.05,20.28) | 0.64(0.26,1.05) | 24.13(9.67,40.67) | 0.59(0.24,1.00) | -0.24(-0.75,0.27) |  |
| Tuvalu | 0.08(0.03,0.13) | 1.10(0.44,1.85) | 0.16(0.07,0.26) | 1.53(0.65,2.50) | 1.09(1.06,1.11) |  |
| Uganda | 16.39(5.35,28.04) | 0.25(0.08,0.44) | 73.63(26.57,126.57) | 0.49(0.17,0.84) | 1.57(1.32,1.82) |  |
| Ukraine | 1565.93(669.11,2428.25) | 2.17(0.93,3.36) | 1698.29(735.96,2893.43) | 2.16(0.93,3.66) | -0.19(-0.33,-0.05) |  |
| United Arab Emirates | 7.88(3.02,14.29) | 1.69(0.64,2.99) | 62.77(25.56,104.32) | 2.25(0.93,3.76) | 2.65(2.09,3.21) |  |
| United Kingdom | 2382.62(1021.75,3890.40) | 2.58(1.11,4.20) | 2882.69(1256.61,4705.18) | 2.08(0.91,3.38) | -0.75(-0.84,-0.67) |  |
| United Republic of Tanzania | 37.31(13.73,61.47) | 0.36(0.13,0.59) | 169.16(65.08,293.62) | 0.70(0.28,1.18) | 2.24(2.19,2.30) |  |
| United States of America | 7377.19(3137.40,11843.81) | 2.28(0.97,3.65) | 11401.66(5070.01,17660.93) | 1.96(0.88,3.03) | -0.69(-0.80,-0.57) |  |
| United States Virgin Islands | 1.95(0.82,3.16) | 2.54(1.05,4.09) | 3.43(1.54,5.53) | 1.96(0.88,3.15) | -0.70(-0.93,-0.47) |  |
| Uruguay | 101.14(41.46,166.87) | 2.59(1.06,4.26) | 201.64(89.26,336.22) | 3.45(1.54,5.72) | 0.81(0.70,0.92) |  |
| Uzbekistan | 73.54(30.51,120.92) | 0.64(0.27,1.07) | 164.78(65.66,278.47) | 0.61(0.24,1.04) | -0.08(-0.50,0.34) |  |
| Vanuatu | 0.41(0.16,0.74) | 0.66(0.25,1.20) | 1.66(0.64,2.78) | 0.94(0.37,1.60) | 1.06(0.98,1.15) |  |
| Venezuela (Bolivarian Republic of) | 85.95(35.49,140.87) | 0.91(0.37,1.50) | 430.13(176.78,724.33) | 1.46(0.60,2.44) | 1.50(1.40,1.61) |  |
| Viet Nam | 34.73(7.83,60.26) | 0.09(0.02,0.15) | 311.39(106.88,520.24) | 0.30(0.10,0.51) | 4.65(4.48,4.81) |  |
| Yemen | 14.68(5.51,26.66) | 0.30(0.11,0.53) | 83.45(28.57,144.27) | 0.60(0.21,1.05) | 2.40(2.27,2.53) |  |
| Zambia | 9.04(3.43,15.50) | 0.31(0.12,0.54) | 62.39(21.76,133.88) | 0.88(0.31,1.78) | 3.29(3.18,3.40) |  |
| Zimbabwe | 17.07(6.57,27.96) | 0.44(0.17,0.72) | 82.59(32.54,138.99) | 1.23(0.49,2.04) | 3.95(3.35,4.55) |  |

**Table S2.DALYs and ASDR of CRC attributable to the high BMI in 1990 and 2021 and the EAPC from 1990 to 2021.**

| DALYs | 1990 |  | 2021 |  | EAPC(1990-2021) |
| --- | --- | --- | --- | --- | --- |
| location | DALYs Cases(95%UI) | ASDR(95%UI) | DALYs Cases(95%UI) | ASDR(95%UI) | ASDR(95%CI) |
| Global | 1015042.12(429787.23,1631973.77) | 25.54(10.83,41.20) | 2364664.16(1021593.57,3752340.44) | 27.33(11.80,43.37) | 0.12(0.08,0.16) |
| Afghanistan | 1787.97(395.41,3506.04) | 23.82(5.40,46.44) | 4014.64(1226.01,7756.27) | 31.38(10.24,59.40) | 0.97(0.87,1.07) |
| Albania | 361.58(147.98,604.30) | 17.52(7.22,29.23) | 874.21(369.42,1483.01) | 20.19(8.57,34.28) | 0.76(0.56,0.97) |
| Algeria | 1033.25(410.03,1763.65) | 8.28(3.30,14.08) | 4690.78(1930.85,7694.87) | 12.87(5.28,21.07) | 1.57(1.44,1.70) |
| American Samoa | 14.30(6.59,22.09) | 53.92(24.52,84.14) | 34.66(16.68,53.86) | 68.05(32.70,104.96) | 0.86(0.76,0.97) |
| Andorra | 28.51(10.59,53.41) | 48.97(18.15,91.52) | 57.02(22.40,102.53) | 37.18(14.60,66.80) | -0.53(-0.79,-0.28) |
| Angola | 263.11(94.42,459.73) | 5.67(2.03,9.95) | 1707.08(628.58,3096.51) | 12.08(4.42,21.79) | 2.43(2.30,2.57) |
| Antigua and Barbuda | 12.08(5.02,19.52) | 23.39(9.65,37.85) | 44.28(19.15,69.52) | 40.51(17.36,63.76) | 1.91(1.75,2.07) |
| Argentina | 16545.41(7128.51,27182.84) | 50.94(21.93,83.66) | 34748.75(15258.18,57059.94) | 63.31(27.85,104.05) | 1.02(0.79,1.25) |
| Armenia | 1151.28(478.29,1813.18) | 39.73(16.45,63.00) | 1695.62(730.21,2731.96) | 39.04(16.78,63.01) | 0.19(0.02,0.36) |
| Australia | 10554.43(4381.52,16830.70) | 54.81(22.76,87.47) | 19249.95(8365.05,30518.39) | 45.46(19.82,71.85) | -0.86(-0.96,-0.76) |
| Austria | 5748.40(2314.23,9248.53) | 50.00(20.15,80.42) | 4949.73(2059.12,8330.21) | 28.38(11.79,47.67) | -1.88(-1.94,-1.81) |
| Azerbaijan | 1418.86(572.90,2305.36) | 26.12(10.56,42.45) | 2788.00(1177.95,4629.45) | 24.45(10.43,40.75) | 0.01(-0.23,0.25) |
| Bahamas | 67.93(28.98,109.50) | 40.33(17.12,65.10) | 269.85(111.54,432.77) | 63.01(25.97,101.27) | 1.76(1.64,1.88) |
| Bahrain | 60.73(25.62,98.31) | 29.29(12.19,47.39) | 383.91(165.38,626.71) | 36.96(15.73,60.07) | 0.27(0.03,0.52) |
| Bangladesh | 903.48(260.86,1602.05) | 1.68(0.49,2.95) | 5088.24(1812.42,9043.76) | 3.41(1.21,6.04) | 2.72(2.54,2.91) |
| Barbados | 114.27(48.24,189.69) | 42.01(17.74,69.27) | 354.86(156.13,583.38) | 70.31(30.90,115.60) | 2.04(1.78,2.30) |
| Belarus | 5529.72(2242.27,9062.41) | 42.25(17.08,69.48) | 9473.97(3919.79,16199.67) | 59.18(24.41,101.14) | 0.35(0.03,0.67) |
| Belgium | 6304.87(2571.65,10166.99) | 41.90(17.18,67.57) | 7389.76(3140.49,12367.33) | 33.13(14.06,54.85) | -0.74(-0.88,-0.61) |
| Belize | 15.72(6.65,25.25) | 16.37(6.92,26.26) | 104.68(46.65,163.57) | 32.36(14.36,50.53) | 2.26(1.76,2.76) |
| Benin | 130.46(48.76,218.44) | 6.18(2.31,10.28) | 546.09(214.72,945.94) | 9.53(3.72,16.28) | 1.50(1.41,1.59) |
| Bermuda | 42.97(17.86,71.21) | 68.09(28.28,112.90) | 81.00(36.11,130.33) | 62.03(27.71,99.29) | -0.15(-0.31,0.01) |
| Bhutan | 21.19(7.38,38.72) | 7.10(2.48,12.91) | 60.52(23.68,110.16) | 9.32(3.61,17.03) | 0.84(0.75,0.92) |
| Bolivia (Plurinational State of) | 807.55(282.58,1488.23) | 23.32(8.23,43.31) | 3353.44(1351.02,5867.85) | 35.17(13.90,61.45) | 1.30(1.27,1.33) |
| Bosnia and Herzegovina | 1402.25(569.77,2295.17) | 32.19(13.10,52.82) | 3262.30(1401.67,5348.90) | 52.91(22.78,86.79) | 1.92(1.67,2.17) |
| Botswana | 70.96(26.70,130.87) | 12.08(4.64,21.89) | 358.54(138.07,621.03) | 23.08(9.17,39.83) | 2.43(2.13,2.74) |
| Brazil | 19032.67(7959.06,30826.94) | 19.90(8.26,32.23) | 92419.74(39036.31,147505.92) | 36.16(15.27,57.77) | 1.87(1.76,1.98) |
| Brunei Darussalam | 39.96(16.63,67.74) | 30.92(12.59,52.09) | 171.09(70.16,281.59) | 39.51(16.05,65.57) | 1.28(1.03,1.52) |
| Bulgaria | 7245.98(3206.28,11789.34) | 58.15(25.65,94.36) | 10795.78(4525.61,18050.10) | 79.88(33.48,133.24) | 1.37(1.21,1.54) |
| Burkina Faso | 133.69(45.30,221.33) | 2.82(0.94,4.69) | 493.96(165.88,877.51) | 4.75(1.59,8.40) | 1.92(1.75,2.08) |
| Burundi | 119.03(35.11,215.04) | 4.76(1.41,8.56) | 316.01(99.33,558.95) | 5.69(1.85,10.17) | 0.10(-0.15,0.36) |
| Cabo Verde | 8.69(3.29,14.49) | 4.01(1.53,6.69) | 69.15(27.24,113.92) | 15.25(6.08,24.95) | 3.86(3.49,4.24) |
| Cambodia | 354.61(103.40,631.47) | 6.75(1.93,11.94) | 1642.57(574.62,2786.41) | 11.70(4.05,19.80) | 1.78(1.68,1.87) |
| Cameroon | 567.59(226.30,932.55) | 11.79(4.70,19.41) | 2776.68(1005.94,4900.32) | 20.22(7.30,35.10) | 1.87(1.78,1.96) |
| Canada | 16298.30(7088.17,26040.66) | 50.72(22.18,80.60) | 28464.63(12799.73,45611.45) | 42.24(19.01,67.63) | -0.45(-0.55,-0.35) |
| Central African Republic | 85.24(29.23,158.15) | 6.28(2.17,11.52) | 333.40(112.03,617.59) | 11.98(4.05,22.16) | 2.13(2.08,2.17) |
| Chad | 105.65(38.58,175.81) | 3.67(1.35,6.05) | 444.15(171.85,740.18) | 7.08(2.80,11.95) | 2.32(2.23,2.42) |
| Chile | 3231.46(1344.53,5236.08) | 31.60(13.15,51.33) | 11318.91(5157.47,18027.95) | 44.67(20.39,71.21) | 1.43(1.30,1.56) |
| China | 109322.47(37789.24,186965.75) | 11.88(4.11,20.31) | 507316.48(209263.93,853770.02) | 24.22(9.98,40.69) | 2.31(2.19,2.42) |
| Colombia | 3323.61(1369.51,5401.29) | 17.72(7.26,28.64) | 17786.94(7455.52,29651.96) | 32.23(13.50,53.71) | 1.82(1.66,1.98) |
| Comoros | 17.55(5.90,30.82) | 7.95(2.64,13.95) | 90.03(32.71,164.26) | 16.97(6.13,30.58) | 2.35(2.20,2.50) |
| Congo | 141.00(51.74,254.10) | 11.62(4.26,20.84) | 657.91(239.13,1130.34) | 19.85(7.37,34.34) | 1.55(1.42,1.69) |
| Cook Islands | 3.58(1.58,5.75) | 26.50(11.60,42.79) | 5.85(2.75,9.46) | 23.45(11.01,37.93) | -0.49(-0.68,-0.30) |
| Costa Rica | 381.09(157.64,616.86) | 20.90(8.63,33.93) | 2711.97(1144.40,4347.78) | 49.16(20.71,78.68) | 3.02(2.80,3.24) |
| Cote d'Ivoire | 245.01(94.52,408.32) | 5.21(2.05,8.69) | 1023.26(375.65,1811.91) | 7.85(2.94,13.68) | 1.36(1.25,1.47) |
| Croatia | 3483.15(1488.47,5721.60) | 57.07(24.29,93.03) | 6433.05(2818.68,10493.31) | 74.39(32.58,120.52) | 0.94(0.71,1.17) |
| Cuba | 2684.86(1097.33,4294.99) | 26.16(10.70,41.85) | 8629.70(3528.98,14288.97) | 45.53(18.61,75.46) | 1.96(1.84,2.08) |
| Cyprus | 202.03(77.85,330.79) | 26.27(10.05,42.56) | 562.25(240.96,946.88) | 27.96(11.94,46.92) | 0.51(0.35,0.67) |
| Czechia | 14060.93(6233.08,22082.20) | 102.03(45.31,159.74) | 13383.68(5790.33,21865.73) | 64.35(27.78,105.58) | -1.81(-1.99,-1.63) |
| Democratic People's Republic of Korea | 1120.66(308.40,2102.67) | 6.64(1.90,12.37) | 3808.02(1291.34,7026.67) | 11.63(3.94,21.44) | 1.94(1.83,2.05) |
| Democratic Republic of the Congo | 843.23(280.83,1468.31) | 4.94(1.64,8.63) | 4345.99(1542.28,7729.37) | 10.71(3.80,19.36) | 2.60(2.36,2.85) |
| Denmark | 3352.19(1392.84,5458.86) | 43.57(18.01,70.80) | 4655.71(1980.67,7815.48) | 40.22(17.16,67.35) | -0.57(-0.85,-0.29) |
| Djibouti | 7.73(2.39,15.11) | 4.61(1.41,8.98) | 75.28(25.19,136.49) | 9.80(3.16,17.44) | 2.42(2.36,2.47) |
| Dominica | 19.18(8.03,29.99) | 32.76(13.76,51.56) | 43.93(20.10,69.25) | 52.07(23.68,81.99) | 1.64(1.56,1.71) |
| Dominican Republic | 457.42(175.59,784.07) | 11.27(4.33,19.35) | 2400.91(974.26,4001.24) | 23.25(9.40,38.73) | 2.79(2.63,2.94) |
| Ecuador | 787.64(328.50,1262.40) | 14.03(5.84,22.49) | 5130.77(2188.36,8366.18) | 30.77(13.08,50.18) | 2.83(2.50,3.17) |
| Egypt | 5362.11(2308.06,8394.06) | 16.84(7.22,26.54) | 27657.51(12311.80,43682.85) | 38.98(17.47,61.19) | 3.35(3.08,3.61) |
| El Salvador | 436.92(182.25,706.55) | 13.81(5.76,22.30) | 1873.21(815.68,3094.06) | 30.67(13.39,50.73) | 2.63(2.44,2.82) |
| Equatorial Guinea | 23.31(8.64,41.18) | 10.51(4.03,18.59) | 151.14(58.41,286.11) | 25.21(9.83,46.93) | 3.00(2.84,3.15) |
| Eritrea | 59.31(20.69,103.50) | 4.22(1.46,7.39) | 289.03(101.73,492.49) | 8.75(3.18,14.90) | 2.43(2.38,2.49) |
| Estonia | 1049.64(430.57,1703.40) | 51.15(21.00,82.78) | 1350.74(588.03,2238.99) | 51.88(22.60,86.17) | -0.27(-0.45,-0.09) |
| Eswatini | 76.84(31.03,133.09) | 25.61(10.23,44.34) | 333.83(128.75,589.93) | 54.84(21.91,95.54) | 2.88(2.24,3.51) |
| Ethiopia | 2719.79(839.60,4642.24) | 12.04(3.71,20.31) | 5678.74(2078.89,9385.58) | 11.85(4.29,19.67) | -0.51(-0.75,-0.26) |
| Fiji | 102.92(42.76,173.85) | 24.65(10.24,41.60) | 293.72(126.00,480.22) | 35.93(15.34,58.54) | 1.30(1.08,1.52) |
| Finland | 2163.31(891.27,3581.72) | 30.79(12.68,51.23) | 3538.78(1526.52,5896.37) | 29.46(12.61,49.59) | -0.20(-0.27,-0.13) |
| France | 30609.24(12509.91,49899.23) | 37.94(15.46,61.62) | 47130.42(19860.23,77738.11) | 35.29(14.96,58.46) | -0.18(-0.28,-0.07) |
| Gabon | 138.22(49.27,267.91) | 23.05(8.21,44.50) | 442.57(180.54,769.94) | 37.86(15.48,66.59) | 1.54(1.43,1.64) |
| Gambia | 14.31(5.09,24.49) | 3.67(1.32,6.28) | 65.79(25.89,118.88) | 6.06(2.39,10.64) | 1.46(1.27,1.66) |
| Georgia | 1841.57(750.83,2894.18) | 29.00(11.82,45.59) | 2218.02(965.34,3621.34) | 39.20(17.05,63.78) | 2.03(1.63,2.44) |
| Germany | 74845.01(31329.34,120489.98) | 60.00(25.06,97.13) | 70676.41(29411.34,118658.52) | 39.25(16.33,65.25) | -1.65(-1.77,-1.53) |
| Ghana | 370.02(143.72,622.20) | 5.15(1.97,8.71) | 2303.22(899.93,3951.05) | 12.59(4.87,21.47) | 3.07(3.01,3.12) |
| Greece | 4468.55(1846.41,7366.66) | 29.67(12.27,48.94) | 8625.12(3544.36,14172.80) | 38.54(16.09,63.31) | 0.60(0.43,0.77) |
| Greenland | 42.87(18.23,70.76) | 114.37(49.19,188.69) | 54.63(22.72,90.02) | 74.53(31.10,122.01) | -1.40(-1.49,-1.32) |
| Grenada | 16.56(6.58,26.90) | 25.13(9.94,40.74) | 51.94(22.26,83.91) | 44.10(18.67,71.60) | 2.18(2.06,2.30) |
| Guam | 35.44(14.87,57.31) | 40.78(16.78,66.40) | 86.42(38.54,136.81) | 42.61(19.03,67.70) | 0.62(0.31,0.93) |
| Guatemala | 413.93(173.77,669.35) | 10.65(4.51,17.30) | 2633.04(1139.38,4363.27) | 22.39(9.70,37.02) | 2.37(2.09,2.66) |
| Guinea | 139.48(51.60,237.25) | 4.01(1.49,6.76) | 403.17(143.70,699.60) | 6.51(2.36,11.15) | 1.67(1.63,1.71) |
| Guinea-Bissau | 33.12(11.56,59.30) | 7.43(2.61,13.29) | 112.87(44.84,197.68) | 13.12(5.22,22.34) | 2.02(1.96,2.08) |
| Guyana | 85.98(33.74,140.87) | 20.97(8.16,34.36) | 249.43(109.92,422.51) | 36.06(15.84,60.70) | 2.12(1.86,2.38) |
| Haiti | 377.72(119.63,679.97) | 10.31(3.27,18.44) | 1478.53(563.09,2612.27) | 17.46(6.68,31.00) | 2.03(1.92,2.14) |
| Honduras | 205.83(78.43,344.58) | 9.12(3.50,15.26) | 1200.86(501.95,2005.77) | 17.89(7.48,30.07) | 2.31(2.14,2.48) |
| Hungary | 12390.17(5137.20,19260.27) | 84.84(35.39,131.42) | 16874.57(7667.73,27562.27) | 92.03(41.88,149.98) | 0.16(-0.09,0.40) |
| Iceland | 105.37(44.63,169.05) | 37.78(16.05,60.60) | 173.70(74.83,290.47) | 30.73(13.29,51.17) | -0.63(-0.74,-0.52) |
| India | 12760.85(4416.44,20302.10) | 2.30(0.79,3.67) | 69114.18(27152.06,108322.44) | 5.35(2.10,8.38) | 2.76(2.67,2.84) |
| Indonesia | 6281.09(2049.99,10670.98) | 5.23(1.64,8.94) | 38734.92(15423.76,66253.59) | 13.84(5.52,23.65) | 3.36(3.14,3.59) |
| Iran (Islamic Republic of) | 3847.30(1627.90,6262.73) | 13.12(5.55,21.38) | 20094.82(8656.32,31960.51) | 24.26(10.42,38.53) | 2.41(2.18,2.63) |
| Iraq | 1513.24(627.50,2457.85) | 17.27(7.19,28.22) | 6317.52(2655.48,10624.90) | 23.42(9.90,39.16) | 1.07(0.90,1.23) |
| Ireland | 2054.52(832.40,3369.68) | 51.93(21.06,85.47) | 2927.82(1254.99,4936.56) | 37.91(16.27,64.06) | -0.90(-1.00,-0.80) |
| Israel | 2124.10(881.75,3369.85) | 44.37(18.42,70.45) | 3847.57(1691.56,6360.96) | 31.73(13.92,52.43) | -1.60(-1.93,-1.27) |
| Italy | 31951.96(12929.68,52382.91) | 36.72(14.80,60.39) | 46935.69(19838.12,76614.84) | 33.89(14.30,55.27) | -0.40(-0.58,-0.23) |
| Jamaica | 353.21(150.72,563.38) | 20.26(8.61,32.39) | 1419.01(631.30,2351.93) | 45.95(20.45,76.16) | 2.75(2.35,3.14) |
| Japan | 32340.37(11926.90,52308.97) | 18.98(6.99,30.67) | 63969.11(24871.05,101820.55) | 20.61(8.17,32.49) | 0.15(0.07,0.23) |
| Jordan | 553.64(235.04,911.14) | 35.76(15.20,58.91) | 3128.39(1340.47,5091.62) | 37.53(16.21,60.86) | 0.12(-0.14,0.38) |
| Kazakhstan | 5330.60(2193.80,8674.88) | 40.02(16.45,65.48) | 6137.89(2668.41,10092.71) | 32.44(14.21,53.24) | -0.46(-0.68,-0.24) |
| Kenya | 455.94(166.38,754.27) | 4.81(1.75,7.91) | 3372.06(1314.25,5703.10) | 12.54(4.87,21.16) | 3.56(3.36,3.75) |
| Kiribati | 11.00(4.46,18.11) | 24.80(9.99,41.00) | 29.11(11.92,47.06) | 33.54(13.93,54.22) | 0.86(0.77,0.94) |
| Kuwait | 131.04(57.38,208.97) | 18.02(7.74,28.40) | 1342.86(618.17,2161.51) | 38.08(17.99,61.08) | 2.75(2.22,3.28) |
| Kyrgyzstan | 951.92(380.83,1562.36) | 30.65(12.25,50.13) | 1255.58(525.77,1973.86) | 23.68(9.90,37.17) | -0.70(-0.83,-0.58) |
| Lao People's Democratic Republic | 186.41(54.37,354.65) | 7.96(2.36,15.08) | 782.43(287.38,1404.58) | 14.49(5.29,26.03) | 2.05(1.92,2.18) |
| Latvia | 1841.34(774.12,2960.81) | 51.50(21.66,82.90) | 1997.64(881.39,3290.21) | 53.27(23.63,87.36) | 0.10(-0.08,0.27) |
| Lebanon | 758.52(285.18,1344.75) | 33.81(12.78,59.23) | 2245.34(929.37,3554.95) | 37.43(15.53,59.19) | 0.74(0.53,0.94) |
| Lesotho | 102.52(39.15,172.58) | 11.89(4.53,20.03) | 419.34(160.69,746.07) | 36.64(14.06,65.03) | 4.70(4.12,5.28) |
| Liberia | 100.77(38.37,166.27) | 8.29(3.12,13.56) | 332.15(111.57,617.96) | 13.43(4.54,24.91) | 1.83(1.60,2.06) |
| Libya | 662.34(284.34,1131.55) | 32.63(14.02,55.32) | 3445.08(1399.84,5748.82) | 58.24(23.43,98.01) | 2.20(2.04,2.36) |
| Lithuania | 1925.95(822.74,3109.86) | 42.76(18.27,68.87) | 2913.99(1281.98,4760.50) | 53.05(23.22,86.13) | 0.64(0.45,0.84) |
| Luxembourg | 284.49(117.34,451.96) | 53.17(21.87,84.37) | 378.43(168.76,610.28) | 35.73(15.89,57.76) | -1.23(-1.43,-1.04) |
| Madagascar | 269.77(92.29,460.23) | 4.91(1.70,8.25) | 1108.43(428.08,1928.54) | 8.62(3.37,15.10) | 1.84(1.66,2.02) |
| Malawi | 124.19(39.57,214.55) | 2.86(0.92,4.92) | 515.10(179.84,929.47) | 6.01(2.14,10.65) | 2.19(2.00,2.38) |
| Malaysia | 2244.43(900.36,3562.14) | 21.69(8.70,34.38) | 10617.07(4396.65,16988.03) | 35.35(14.64,56.32) | 1.42(1.27,1.58) |
| Maldives | 8.45(2.60,15.01) | 7.38(2.33,13.11) | 29.48(11.67,50.19) | 6.89(2.70,11.64) | -0.52(-0.64,-0.40) |
| Mali | 257.16(94.59,427.80) | 5.86(2.10,9.71) | 777.04(284.59,1358.97) | 7.81(2.82,13.53) | 1.05(0.90,1.20) |
| Malta | 133.61(53.65,215.46) | 31.27(12.57,50.24) | 310.08(133.53,524.89) | 33.44(14.43,56.28) | 0.16(0.03,0.29) |
| Marshall Islands | 6.74(3.01,11.42) | 35.76(15.90,60.63) | 20.08(8.44,33.72) | 47.59(20.08,79.12) | 0.89(0.81,0.96) |
| Mauritania | 120.07(48.07,202.39) | 11.73(4.64,19.89) | 388.46(148.56,660.93) | 17.58(6.69,29.73) | 1.30(1.15,1.46) |
| Mauritius | 96.35(39.63,155.80) | 12.12(5.01,19.73) | 632.94(268.79,1015.51) | 34.35(14.56,54.95) | 2.87(2.66,3.07) |
| Mexico | 6822.99(2890.42,10947.31) | 14.77(6.26,23.75) | 44172.92(19788.52,70036.06) | 33.34(14.91,52.92) | 2.71(2.59,2.84) |
| Micronesia (Federated States of) | 21.13(8.50,36.21) | 39.38(15.79,68.20) | 42.68(17.26,72.04) | 49.93(20.17,83.70) | 0.76(0.72,0.80) |
| Monaco | 37.47(14.58,61.81) | 58.05(22.46,94.96) | 64.31(28.91,109.02) | 71.56(32.28,121.83) | 0.78(0.67,0.90) |
| Mongolia | 207.51(85.45,344.41) | 18.30(7.58,30.36) | 573.82(230.99,964.03) | 21.67(8.73,36.53) | 0.39(0.24,0.53) |
| Montenegro | 266.22(114.24,427.95) | 41.70(17.99,66.63) | 581.94(270.72,953.24) | 59.44(27.63,97.27) | 1.29(1.18,1.40) |
| Morocco | 1855.79(648.54,3181.19) | 12.13(4.26,20.71) | 9012.08(3431.46,16033.25) | 24.71(9.44,44.24) | 2.54(2.44,2.64) |
| Mozambique | 114.32(36.78,196.11) | 1.83(0.59,3.16) | 521.39(189.83,900.24) | 4.34(1.58,7.52) | 3.32(3.12,3.52) |
| Myanmar | 2267.95(760.45,4446.15) | 8.43(2.83,16.57) | 6592.56(2431.49,11314.28) | 12.21(4.50,21.12) | 1.02(0.88,1.15) |
| Namibia | 55.28(22.09,90.50) | 7.87(3.11,12.74) | 246.06(98.03,415.64) | 16.33(6.64,27.35) | 2.28(1.97,2.60) |
| Nauru | 3.96(1.43,7.14) | 67.99(24.83,122.52) | 5.62(2.23,9.67) | 80.07(30.96,138.90) | 0.45(0.41,0.50) |
| Nepal | 258.22(80.53,467.48) | 2.23(0.66,4.08) | 1201.11(429.53,2091.14) | 4.65(1.65,8.10) | 2.68(2.24,3.12) |
| Netherlands | 8868.51(3719.95,14575.62) | 45.17(18.92,74.23) | 16477.88(7036.36,26890.26) | 48.70(20.88,79.43) | 0.36(0.16,0.55) |
| New Zealand | 2869.62(1196.64,4663.19) | 75.04(31.28,121.63) | 4315.48(1819.80,6993.57) | 53.05(22.47,85.87) | -1.23(-1.32,-1.13) |
| Nicaragua | 201.92(83.26,323.91) | 11.78(4.85,18.94) | 1083.28(471.09,1745.38) | 20.78(9.05,33.59) | 2.24(1.98,2.50) |
| Niger | 120.44(41.68,204.97) | 3.83(1.32,6.44) | 476.72(176.39,856.70) | 5.27(1.94,9.36) | 1.11(1.03,1.19) |
| Nigeria | 2583.30(1032.87,4350.83) | 5.65(2.27,9.52) | 10990.43(4367.68,18180.33) | 11.40(4.58,18.66) | 2.42(2.34,2.50) |
| Niue | 0.69(0.30,1.17) | 32.76(14.30,55.70) | 0.98(0.45,1.57) | 45.68(21.09,73.34) | 0.92(0.87,0.97) |
| North Macedonia | 796.51(331.52,1267.98) | 41.26(17.21,65.45) | 1827.08(809.76,3099.98) | 54.69(24.30,92.87) | 0.97(0.66,1.28) |
| Northern Mariana Islands | 12.32(5.14,21.17) | 49.88(21.74,82.36) | 36.14(16.86,56.93) | 62.96(29.41,99.91) | 0.81(0.57,1.04) |
| Norway | 2850.85(1192.16,4598.07) | 44.50(18.66,71.90) | 3597.10(1563.54,5846.10) | 36.77(15.95,59.55) | -0.74(-0.84,-0.63) |
| Oman | 64.49(24.74,112.07) | 8.12(3.12,14.03) | 313.68(124.53,506.48) | 13.22(5.33,20.92) | 2.06(1.76,2.36) |
| Pakistan | 2750.04(943.45,4530.72) | 4.47(1.52,7.39) | 16036.19(6611.20,26630.82) | 11.25(4.56,18.60) | 3.05(2.79,3.32) |
| Palau | 4.69(1.90,7.88) | 45.04(18.06,76.30) | 11.05(4.73,17.77) | 47.81(20.61,77.77) | 0.28(0.19,0.36) |
| Palestine | 465.65(194.48,811.29) | 51.21(21.39,88.66) | 1606.62(657.08,2522.63) | 57.87(23.51,91.04) | 0.46(0.33,0.59) |
| Panama | 303.93(123.36,494.08) | 20.33(8.24,32.91) | 1720.03(756.29,2834.08) | 38.99(17.15,64.25) | 2.40(2.30,2.51) |
| Papua New Guinea | 134.75(50.20,255.76) | 5.88(2.19,11.30) | 477.34(186.85,828.28) | 7.07(2.71,12.26) | 0.49(0.42,0.55) |
| Paraguay | 308.71(126.49,504.55) | 13.29(5.44,21.86) | 1884.35(822.52,3219.35) | 31.34(13.65,53.51) | 3.09(2.90,3.28) |
| Peru | 2079.26(843.39,3474.54) | 16.30(6.56,27.26) | 8463.15(3669.68,14676.05) | 24.74(10.70,42.99) | 1.23(1.04,1.42) |
| Philippines | 3516.59(1293.06,5589.33) | 9.68(3.49,15.40) | 19795.95(7972.48,31958.09) | 21.19(8.55,34.07) | 2.68(2.53,2.82) |
| Poland | 23666.98(10126.72,38511.78) | 53.79(23.08,87.61) | 47994.34(20263.62,78055.74) | 67.69(28.52,109.90) | 0.62(0.43,0.81) |
| Portugal | 5619.79(2316.64,9390.61) | 41.43(17.10,68.96) | 10132.13(4436.53,16698.99) | 44.92(19.61,74.74) | 0.30(0.01,0.59) |
| Puerto Rico | 1425.97(613.49,2281.13) | 39.79(17.12,63.80) | 3231.04(1480.01,5086.84) | 53.20(24.68,82.99) | 0.90(0.70,1.11) |
| Qatar | 54.57(23.50,88.96) | 38.17(16.88,61.50) | 521.48(226.53,865.69) | 43.47(19.13,72.23) | 0.68(0.09,1.28) |
| Republic of Korea | 3813.01(1265.52,6397.36) | 11.58(3.81,19.39) | 14548.13(5639.22,23664.40) | 15.77(6.11,25.56) | 0.87(0.64,1.10) |
| Republic of Moldova | 2331.64(1043.88,3739.10) | 50.85(22.80,81.31) | 4011.68(1746.37,6352.27) | 67.08(29.17,106.44) | 1.37(0.96,1.78) |
| Romania | 9553.22(4025.60,15344.66) | 33.49(14.13,54.13) | 23616.78(9680.46,39634.85) | 67.12(27.77,111.95) | 1.98(1.74,2.22) |
| Russian Federation | 85206.83(36922.86,135435.30) | 46.35(19.97,73.53) | 147445.79(63131.18,233862.53) | 61.98(26.58,98.26) | 0.72(0.54,0.91) |
| Rwanda | 241.00(75.21,434.70) | 7.40(2.30,13.31) | 674.27(218.00,1193.90) | 9.43(3.02,16.83) | -0.11(-0.50,0.28) |
| Saint Kitts and Nevis | 12.11(4.88,19.74) | 34.10(13.75,55.51) | 34.66(15.32,55.55) | 47.85(20.82,77.22) | 1.55(1.39,1.71) |
| Saint Lucia | 18.00(7.18,29.17) | 20.63(8.21,33.32) | 66.80(28.69,109.29) | 27.68(11.93,45.47) | 0.80(0.63,0.96) |
| Saint Vincent and the Grenadines | 11.35(4.48,18.56) | 16.00(6.34,26.00) | 41.81(17.89,68.22) | 29.36(12.49,47.72) | 1.97(1.83,2.11) |
| Samoa | 29.22(12.78,45.60) | 31.70(13.72,49.61) | 62.03(28.48,101.97) | 39.94(18.24,65.53) | 0.74(0.66,0.81) |
| San Marino | 17.13(6.88,28.27) | 49.34(19.77,81.03) | 21.38(8.28,38.32) | 30.05(11.37,53.80) | -0.72(-1.06,-0.38) |
| Sao Tome and Principe | 5.72(2.16,9.64) | 8.76(3.30,14.74) | 22.30(8.97,36.25) | 18.96(7.63,30.71) | 2.54(2.47,2.61) |
| Saudi Arabia | 1114.81(435.76,1923.39) | 15.66(6.27,26.92) | 9497.01(4086.09,15242.00) | 35.03(15.51,55.19) | 2.88(2.49,3.27) |
| Senegal | 224.97(85.65,378.35) | 6.48(2.43,10.97) | 911.56(326.83,1571.59) | 11.03(4.00,18.69) | 1.86(1.69,2.03) |
| Serbia | 6351.05(2805.30,10698.27) | 55.92(24.72,94.35) | 11053.12(5048.51,17655.09) | 68.62(31.39,110.48) | 0.55(0.40,0.71) |
| Seychelles | 17.41(6.66,27.67) | 30.91(11.82,49.19) | 71.79(31.95,113.52) | 57.95(25.73,92.16) | 2.17(1.89,2.45) |
| Sierra Leone | 82.59(27.05,144.00) | 3.93(1.27,6.91) | 312.09(111.62,552.29) | 7.58(2.65,13.33) | 2.47(2.33,2.60) |
| Singapore | 492.25(182.46,816.17) | 20.20(7.32,33.56) | 1913.03(800.10,3104.70) | 21.98(9.18,35.55) | 0.06(-0.17,0.29) |
| Slovakia | 5020.53(2205.10,8198.16) | 83.56(36.72,135.90) | 7788.11(3544.60,12498.85) | 82.15(37.40,131.78) | -0.08(-0.18,0.02) |
| Slovenia | 1453.32(618.73,2348.13) | 58.70(24.95,94.72) | 2088.34(910.15,3457.21) | 48.19(20.93,79.29) | -0.85(-1.16,-0.54) |
| Solomon Islands | 29.09(10.89,52.42) | 17.63(6.44,31.84) | 111.35(41.59,191.94) | 25.97(9.60,44.04) | 1.27(1.12,1.42) |
| Somalia | 247.10(74.51,469.92) | 7.60(2.31,14.31) | 917.01(319.41,1696.52) | 11.52(3.95,21.38) | 1.41(1.35,1.46) |
| South Africa | 4701.63(2021.39,7304.19) | 20.97(9.02,32.96) | 18072.59(7606.08,28074.05) | 36.99(15.66,57.50) | 2.04(1.82,2.27) |
| South Sudan | 121.77(34.15,246.42) | 4.44(1.24,8.99) | 354.31(111.79,643.84) | 7.70(2.43,13.78) | 1.80(1.55,2.05) |
| Spain | 23713.30(10094.07,39207.19) | 44.62(19.03,73.55) | 45826.94(20076.80,75032.91) | 49.15(21.51,79.96) | 0.29(0.09,0.48) |
| Sri Lanka | 499.22(185.05,805.40) | 4.15(1.54,6.72) | 1892.17(758.90,3396.59) | 6.89(2.76,12.41) | 1.91(1.71,2.11) |
| Sudan | 1375.82(511.76,2501.03) | 13.33(5.08,24.11) | 5397.62(2161.42,9731.52) | 23.20(9.41,41.24) | 1.88(1.79,1.98) |
| Suriname | 50.71(19.31,84.45) | 18.74(7.10,31.02) | 209.60(89.10,351.16) | 32.07(13.58,53.70) | 1.98(1.78,2.19) |
| Sweden | 5224.66(2157.48,8654.76) | 36.84(15.32,60.53) | 6588.71(2835.17,10856.52) | 31.99(13.77,53.10) | -0.28(-0.44,-0.12) |
| Switzerland | 2834.87(1185.00,4550.57) | 28.15(11.81,45.26) | 3827.17(1595.83,6321.47) | 21.67(9.15,35.57) | -0.96(-1.11,-0.80) |
| Syrian Arab Republic | 1067.77(428.90,1754.75) | 18.01(7.24,29.51) | 3821.02(1547.18,6399.24) | 27.36(11.00,45.77) | 1.21(1.01,1.41) |
| Taiwan (Province of China) | 3435.32(1327.07,5477.53) | 20.09(7.72,32.28) | 18227.36(7648.34,29381.26) | 44.71(18.68,71.98) | 2.27(1.97,2.56) |
| Tajikistan | 592.10(244.37,942.44) | 20.16(8.34,32.08) | 972.57(399.48,1689.46) | 14.26(5.84,24.55) | -1.18(-1.37,-0.99) |
| Thailand | 4450.49(1610.37,7497.94) | 10.89(3.89,18.17) | 29222.65(11515.65,49985.38) | 27.82(10.96,47.53) | 2.82(2.62,3.03) |
| Timor-Leste | 8.62(2.08,16.03) | 2.35(0.53,4.39) | 47.45(15.17,82.67) | 5.16(1.64,8.96) | 2.78(2.44,3.11) |
| Togo | 67.67(25.20,112.88) | 4.92(1.81,8.21) | 436.65(153.09,774.35) | 10.36(3.58,18.25) | 2.58(2.50,2.66) |
| Tokelau | 0.41(0.18,0.70) | 30.60(13.50,52.86) | 0.56(0.25,0.91) | 38.71(17.27,62.32) | 0.70(0.67,0.72) |
| Tonga | 14.75(6.40,22.67) | 24.58(10.66,38.05) | 25.69(12.07,40.62) | 31.17(14.60,49.11) | 0.71(0.60,0.81) |
| Trinidad and Tobago | 276.48(119.77,432.44) | 32.26(13.96,50.44) | 907.39(384.84,1491.32) | 47.05(19.99,77.48) | 1.13(0.99,1.26) |
| Tunisia | 723.93(296.23,1227.27) | 13.72(5.63,23.22) | 3162.80(1323.12,5584.23) | 23.20(9.69,40.68) | 1.58(1.52,1.64) |
| Turkey | 16374.85(6758.56,26442.80) | 43.43(17.77,70.48) | 44972.27(19098.81,72721.24) | 47.00(19.95,76.08) | 0.10(-0.25,0.46) |
| Turkmenistan | 371.14(150.84,606.19) | 17.65(7.16,28.95) | 726.77(291.97,1219.48) | 16.23(6.53,27.24) | -0.28(-0.78,0.23) |
| Tuvalu | 2.34(0.95,3.94) | 31.28(12.85,52.58) | 4.59(1.94,7.47) | 41.53(17.55,67.22) | 0.93(0.90,0.96) |
| Uganda | 485.62(159.27,834.14) | 6.80(2.24,11.70) | 2304.36(848.66,4017.27) | 13.08(4.74,22.50) | 1.52(1.25,1.80) |
| Ukraine | 40024.09(17028.58,63165.94) | 55.60(23.77,87.72) | 41529.99(18071.94,70692.42) | 54.94(23.80,93.12) | -0.31(-0.46,-0.15) |
| United Arab Emirates | 280.08(108.02,506.31) | 43.30(16.47,77.72) | 2201.58(903.19,3664.32) | 47.03(19.27,77.93) | 1.58(1.14,2.03) |
| United Kingdom | 50520.12(21642.71,81840.55) | 58.29(24.98,94.29) | 55978.14(24503.18,90585.82) | 45.54(19.94,73.27) | -0.87(-0.95,-0.78) |
| United Republic of Tanzania | 1087.62(396.95,1828.73) | 9.19(3.38,15.36) | 4790.20(1827.61,8189.88) | 16.98(6.50,29.33) | 2.01(1.95,2.07) |
| United States of America | 168851.45(72922.94,268750.94) | 54.89(23.79,87.29) | 268295.65(122072.77,412890.81) | 50.36(23.01,77.22) | -0.41(-0.51,-0.32) |
| United States Virgin Islands | 52.26(21.97,84.01) | 59.14(24.79,95.46) | 76.89(34.92,124.19) | 48.30(22.02,77.37) | -0.47(-0.67,-0.26) |
| Uruguay | 2293.67(954.34,3774.98) | 59.95(24.94,98.56) | 4099.14(1831.54,6732.55) | 78.34(35.02,128.15) | 0.78(0.67,0.89) |
| Uzbekistan | 2146.59(891.88,3498.45) | 17.77(7.40,29.11) | 5024.95(2019.86,8463.07) | 16.74(6.69,28.30) | -0.19(-0.62,0.24) |
| Vanuatu | 13.25(5.12,24.25) | 17.98(6.87,32.41) | 52.98(20.49,89.55) | 25.85(10.03,43.32) | 1.05(0.95,1.16) |
| Venezuela (Bolivarian Republic of) | 2383.17(994.57,3864.20) | 22.73(9.48,36.92) | 11354.69(4692.57,19168.65) | 37.08(15.30,62.51) | 1.56(1.44,1.67) |
| Viet Nam | 990.81(231.57,1703.94) | 2.36(0.56,4.07) | 9111.95(3134.10,15453.63) | 8.32(2.86,14.01) | 4.74(4.53,4.95) |
| Yemen | 458.11(170.05,834.68) | 8.15(3.06,14.71) | 2509.34(856.44,4396.14) | 15.40(5.28,26.44) | 2.17(2.06,2.29) |
| Zambia | 283.46(106.79,483.94) | 8.53(3.25,14.62) | 1999.90(680.13,4465.00) | 23.55(8.21,50.42) | 3.19(3.08,3.30) |
| Zimbabwe | 480.93(186.38,788.85) | 10.98(4.23,18.07) | 2485.03(988.21,4198.24) | 31.43(12.39,52.55) | 4.11(3.42,4.81) |
