# Supplementary material for: Analysis of the burden of colorectal cancer attributable to high body mass index in 204 countries and regions worldwide from 1990 to 2021
Source: Front Nutr. 2025 Jun 9;12:1589250. doi: 10.3389/fnut.2025.1589250 (PMC12183065; doi:10.3389/fnut.2025.1589250)
Supplement: Supplementary file 4 [file Image_1.pdf]

Supplementary Material

Supplementary file 1: Supplementary figures

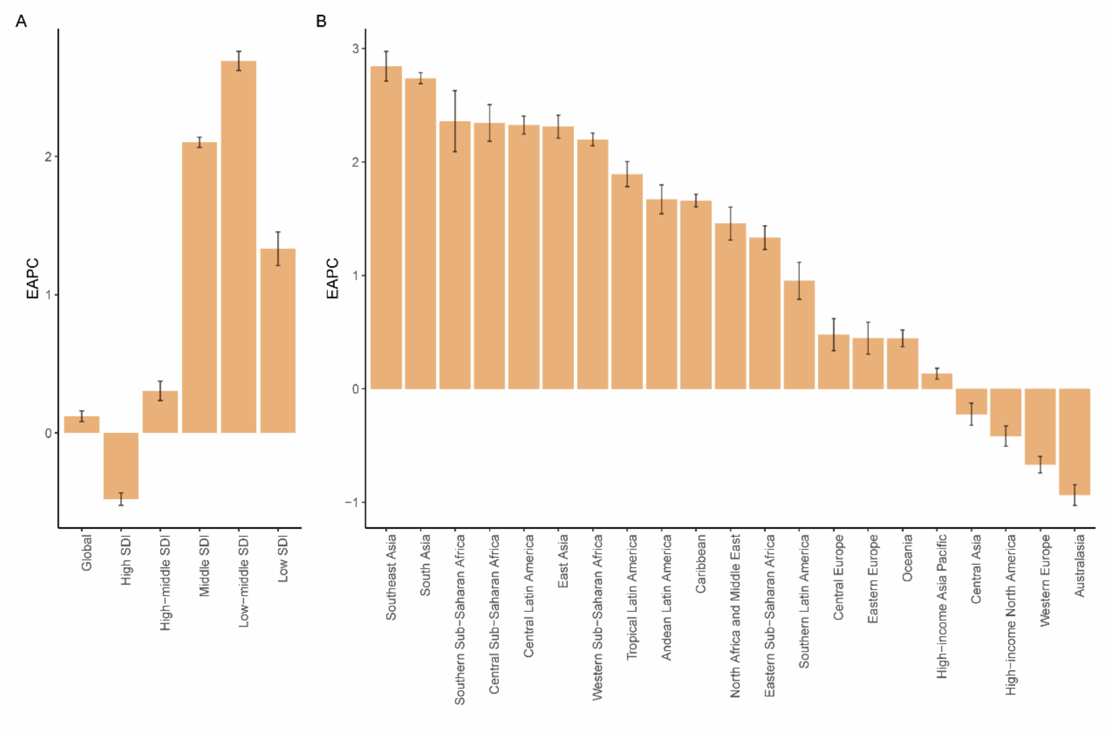

Figure S1.EAPC in ASDR of CRC attributable to high BMI across 5 SDI regions and 21 GBD regions, 1990-2021.

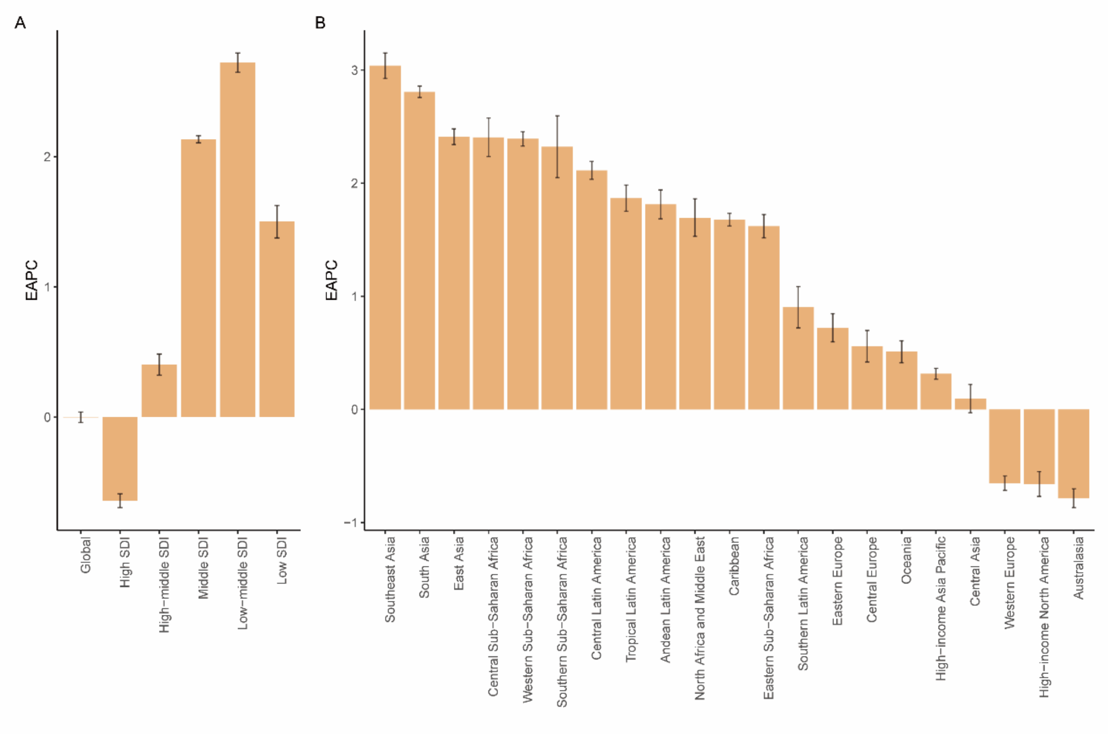

Figure S2.EAPC in ASMR of CRC attributable to high BMI across 5 SDI regions and 21 GBD regions, 1990-2021.

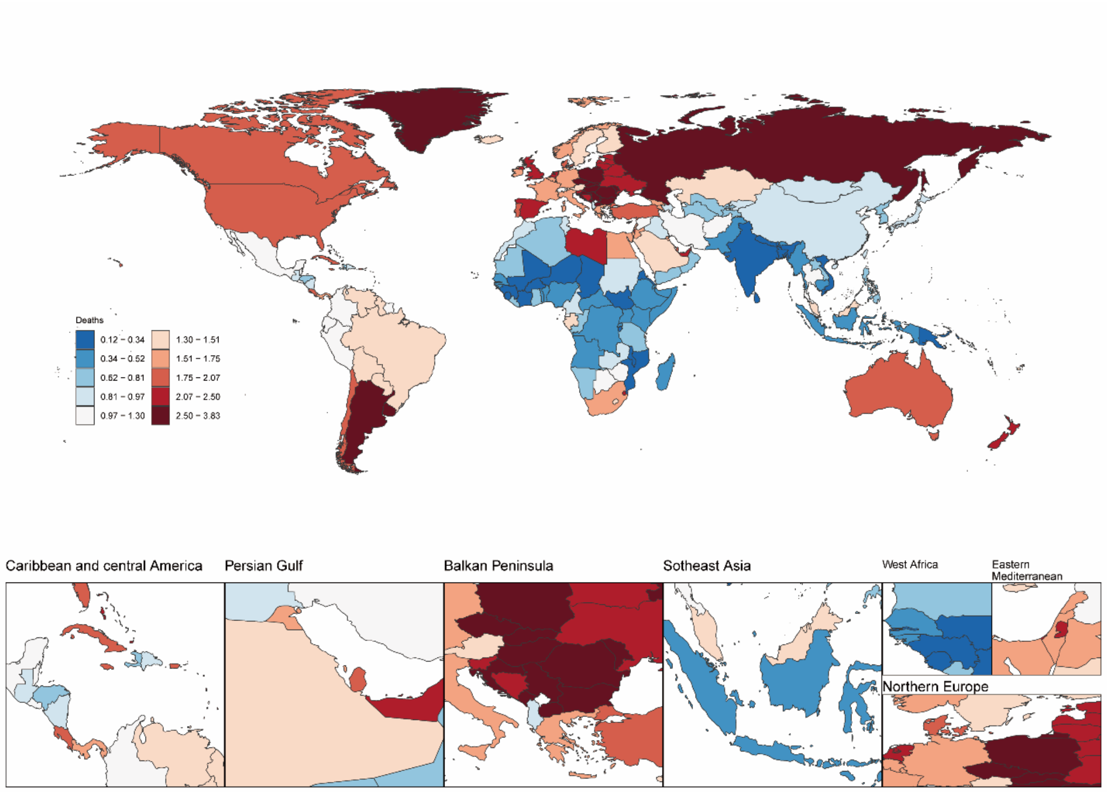

Figure S3. Age-standardized mortality rates (ASMR) of CRC attributable to high BMI per 100,000 population by country/region in 2021.

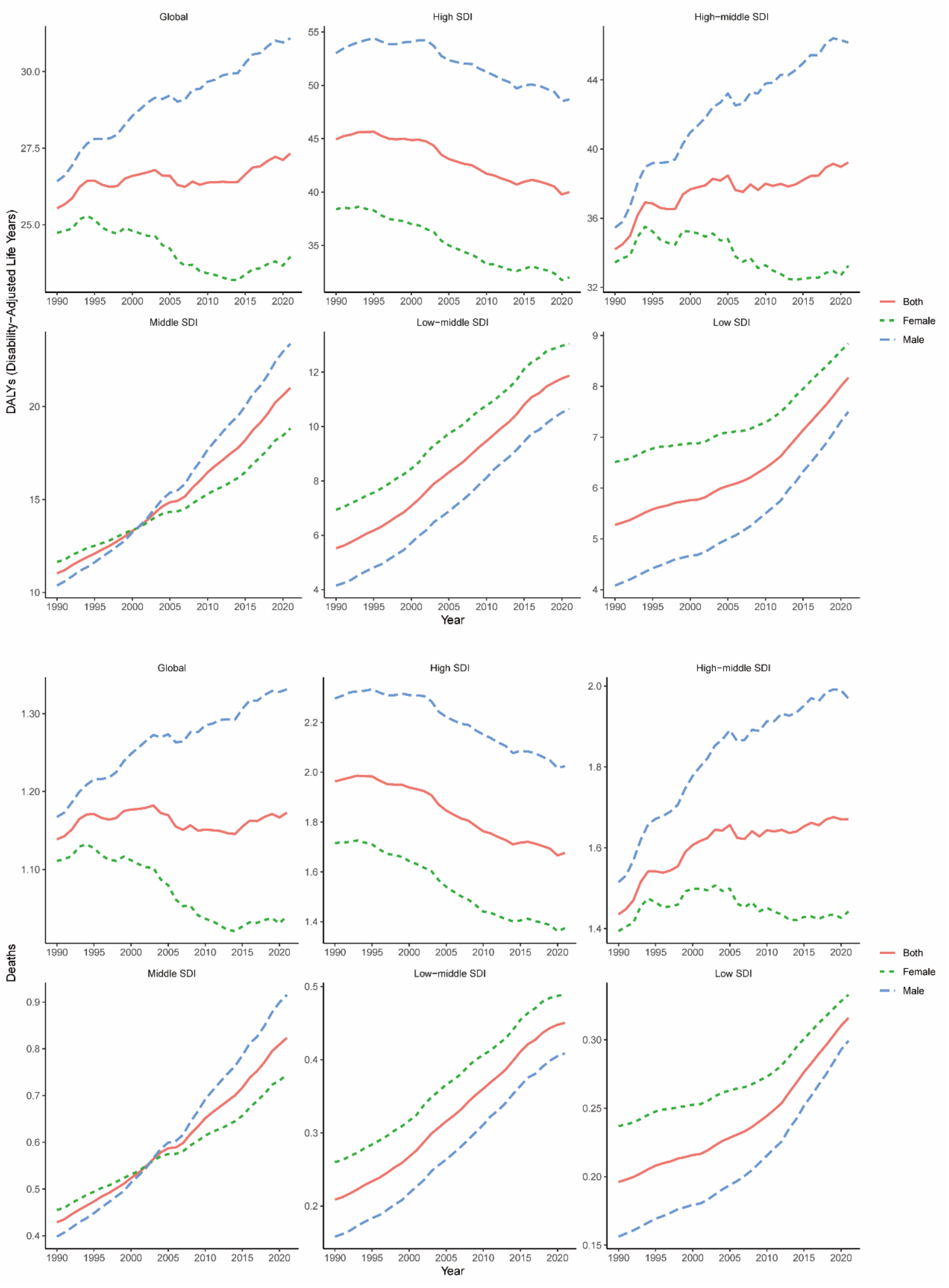

Figure S4. Trends in ASMR and ASDR of CRC attributable to high BMI by sex at global and five SDI region levels, 1990-2021.

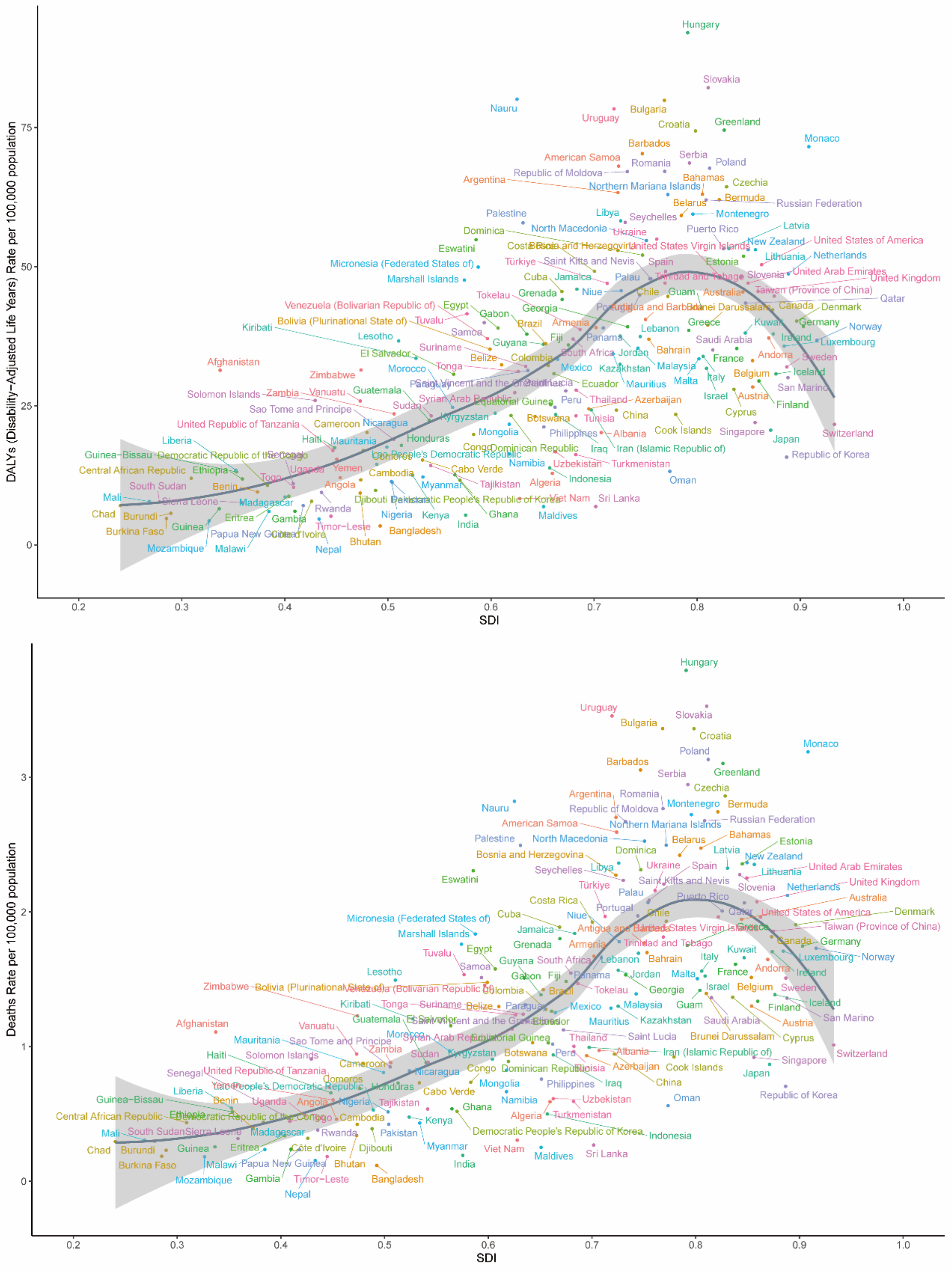

Figure S5. Age-standardized DALY rate and ASMR of CRC attributable to high BMI by SDI across 204 countries and territories globally, 1990-2021.

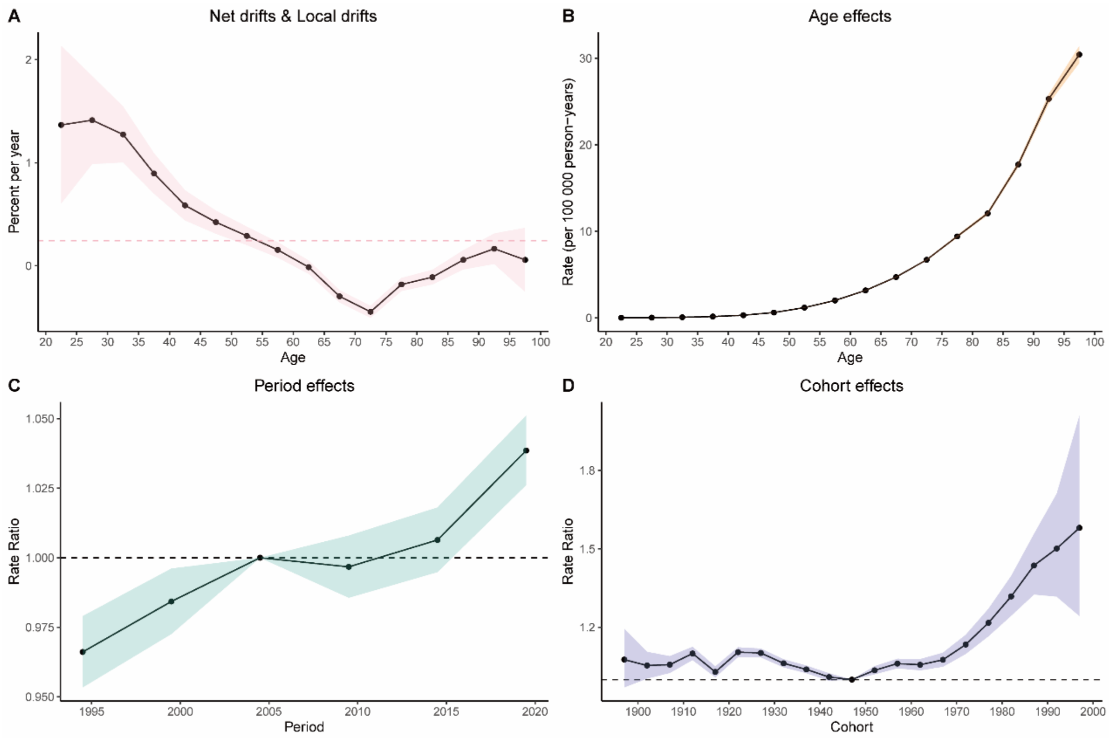

Figure S6. Results of the age-period-cohort analysis:(A) Net drift versus actual drift of mortality rates;(B) Age effects on mortality rates;(C) Period effects on mortality rates;(D) Cohort effects on mortality rates.

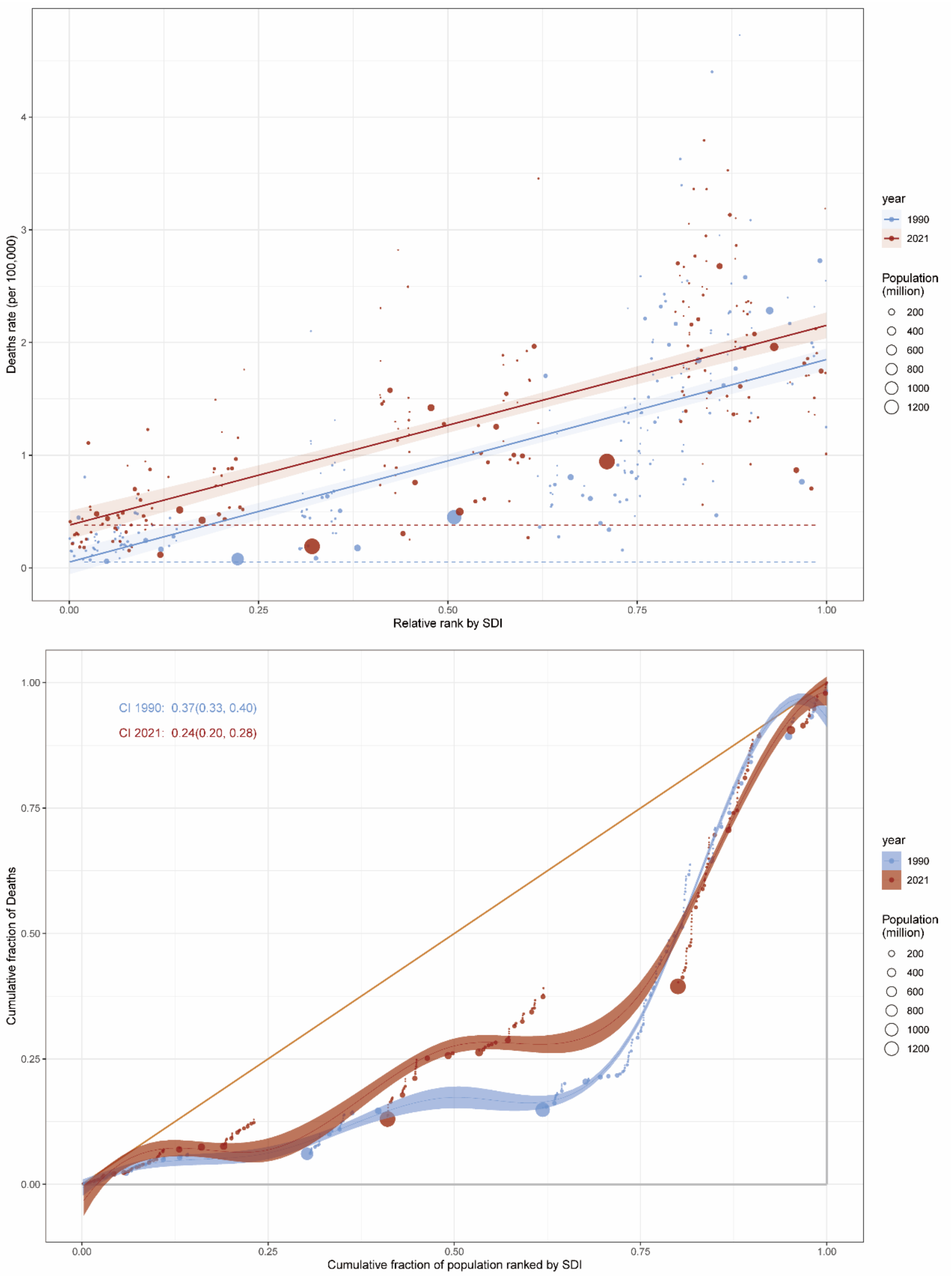

Figure S7. Inequality analysis of mortality attributable to high BMI in CRC at the global level in 1990 and 2021.

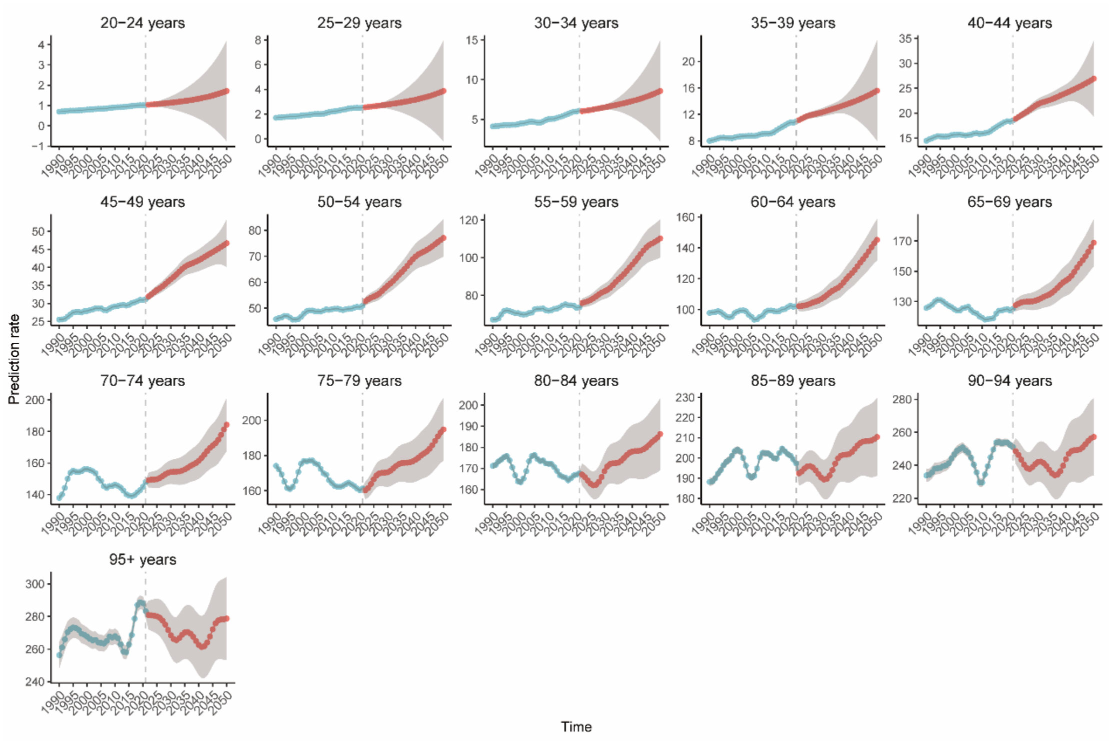

Figure S8 . Temporal trends in DALY rates for CRC attributable to high BMI at the global level from 1990 to 2050.

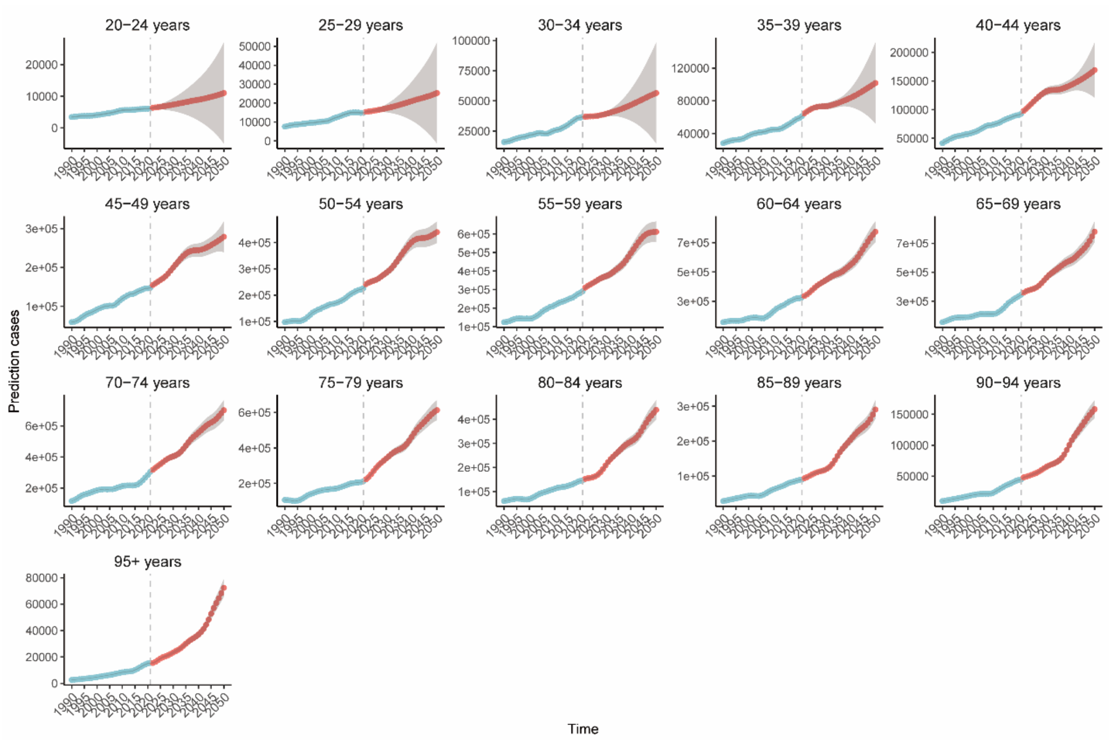

Figure S9. Temporal trends in DALY numbers for CRC attributable to high BMI at the global level from 1990 to 2050.

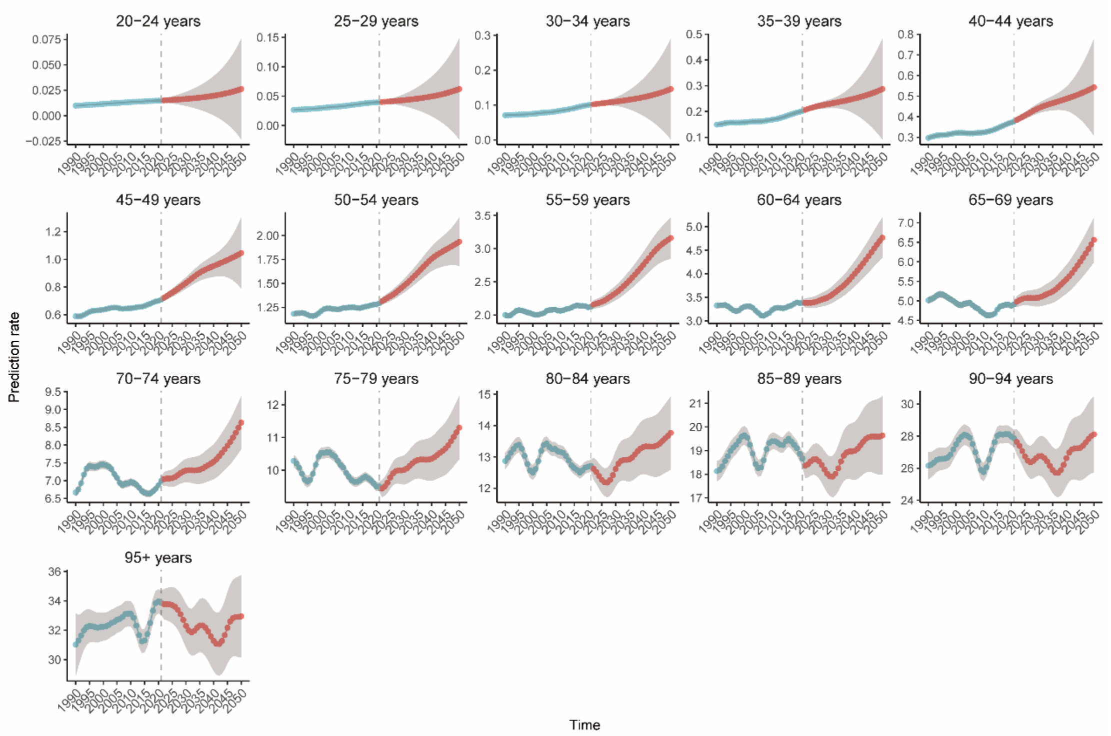

Figure S10. Temporal trends in age-specific mortality rates for CRC attributable to high BMI at the global level from 1990 to 2050.

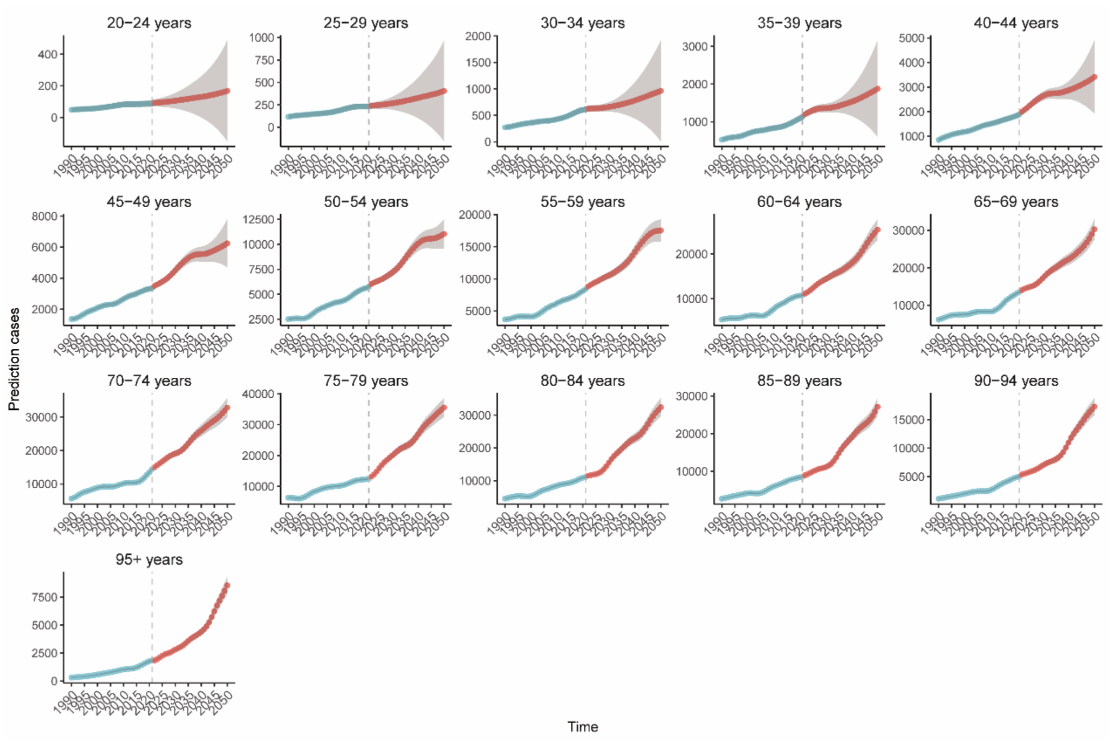

Figure S11. Temporal trends in age-specific death numbers for CRC attributable to high BMI at the global level from 1990 to 2050.
